# Recent Advances in the Characterization of Food Biomacromolecules: Polysaccharide, Protein, and Lipid

**DOI:** 10.1002/fsn3.70523

**Published:** 2025-06-29

**Authors:** Seid Reza Falsafi, Julia Sebastian, Rosana Colussi, Lucas Ávila do Nascimento, Tansel Kemerli‐Kalbaran, Meral Yildirim‐Yalcin, Ivan Adrian Garcia‐Galicia, Selin Sahin, Alma Delia Alarcon‐Rojo

**Affiliations:** ^1^ Food Science and Technology Division, Agricultural Engineering Research Department Safiabad Agricultural and Natural Resources Research and Education Center (AREEO) Dezful Iran; ^2^ ICMR‐National Institute of Nutrition Hyderabad India; ^3^ Center for Pharmaceutical and Food Chemical Sciences Federal University of Pelotas Pelotas Brazil; ^4^ Department of Agroindustrial Science and Technology Federal University of Pelotas Pelotas Brazil; ^5^ Chemical Engineering Department, Engineering Faculty Gebze Technical University Gebze Turkey; ^6^ Food Engineering Department, Engineering Faculty Istanbul Aydin University Istanbul Turkey; ^7^ CEIEGT, Facultad de Medicina Veterinaria y Zootecnia Universidad Nacional Autónoma de México Tlapacoyan Mexico; ^8^ Faculty of Engineering, Chemical Engineering Department, Division of Unit Operations and Thermodynamics Istanbul University‐Cerrahpaşa Istanbul Turkey; ^9^ Facultad de Zootecnia y Ecología Universidad Autónoma de Chihuahua Chihuahua Mexico

**Keywords:** characterization technique, food biomacromolecules, lipid, polysaccharide, protein

## Abstract

Polysaccharides, proteins, and lipids are indispensable biomacromolecules that underpin the structure, functionality, and nutritional quality of food systems. Their multifaceted roles, from imparting stability and texture to enabling bioactive functions, are pivotal in the formulation of innovative and sustainable food products. Yet, their structural complexity, diverse conformations, and dynamic behaviors present formidable challenges in characterization. Recent breakthroughs in advanced analytical techniques—spanning spectroscopic, chromatographic, and imaging modalities—have revolutionized our ability to probe these molecules at unprecedented resolutions. Tools such as multidimensional nuclear magnetic resonance, cutting‐edge mass spectrometry, and state‐of‐the‐art microscopy have illuminated the intricate branching of polysaccharides, the conformational dynamics of proteins, and the amphiphilic properties of lipids. This review delves into these technological advancements, explores their transformative impact on understanding food biomacromolecules, and addresses the enduring challenges posed by heterogeneous food matrices. By shaping future directions, this work aims to foster innovation and expand the scientific understanding of food systems.

## Graphical abstract

Polysaccharides, proteins, and lipids are key biomacromolecules that determine the structure, function, and nutritional value of food systems. Recent advances in spectroscopic, chromatographic, and imaging techniques have significantly enhanced our ability to characterize their complex structures and dynamic behaviors. This review highlights these analytical breakthroughs and their impact on understanding food macromolecules within diverse and heterogeneous matrices.

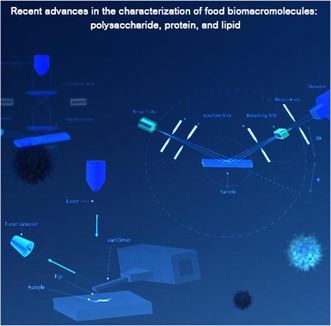

## 1 Introduction

Food biomacromolecules—polysaccharides, proteins, and lipids—serve as fundamental building blocks of food systems, contributing to their texture, stability, and nutritional value. These macromolecules are central to the design and functionality of food products, influencing key properties such as viscosity, gelation, emulsification, and foaming (Mulla et al. 2023). Polysaccharides, for instance, provide structural integrity and water‐binding capacity, while proteins contribute to textural properties and are integral to enzymatic and bioactive functions. Lipids, on the other hand, are crucial for flavor delivery and energy density while also forming the basis of many colloidal systems. Understanding the intricate structure–function relationships of these biomolecules is essential for optimizing their roles in food systems, particularly as the demand for high‐performance, sustainable, and health‐focused food products continues to grow. However, the heterogeneity, and dynamic behavior of these macromolecules add layers of complexity to their analysis. Advanced characterization techniques are, therefore, indispensable for unraveling these complexities and driving innovation in food science and technology (Falsafi et al. 2023).

Polysaccharides, with their diverse branching patterns, varying degrees of polymerization, and susceptibility to extraction conditions, require specialized techniques to elucidate their molecular structure and functional roles in food systems. Proteins, characterized by intricate folding, conformational variability, and sensitivity to denaturation, pose unique challenges in understanding their interactions and stability in complex matrices (Mulla et al. 2023). Lipids, with their amphiphilic behavior, diverse chain structures, and phase transitions, further complicate the analytical landscape, particularly when studied in emulsions and multilayered systems. Recent advances in analytical technologies, including spectroscopic, chromatographic, and microscopic methods, have provided unprecedented opportunities to unravel the structural and functional properties of these biomolecules with remarkable precision (Shahidi et al. 2017). Innovations such as multidimensional nuclear magnetic resonance (NMR), advanced mass spectrometry (MS), and high‐resolution imaging techniques have enabled researchers to probe molecular architecture, interactions, and dynamics at resolutions previously unattainable. These advancements are pivotal for addressing long‐standing challenges in the field while also uncovering novel applications of food biomacromolecules (Kurzyna‐Szklarek et al. 2022).

This review aims to provide a comprehensive overview of recent advances in the characterization of polysaccharides, proteins, and lipids, emphasizing their structural, conformational, and functional analysis in food systems. By synthesizing cutting‐edge developments in spectroscopic, chromatographic, and imaging technologies, as well as emerging integrative approaches, this work seeks to bridge the gap between molecular‐level insights and practical applications. Understanding these biomacromolecules through the lens of advanced analytical tools is essential for addressing critical challenges in food science, including the development of sustainable ingredients, enhancement of food quality, and creation of novel functional products. By highlighting current knowledge and identifying areas requiring further investigation, this review contributes to the growing body of research that underpins innovation in food systems, paving the way for future advancements in both academia and industry.

## 2 Polysaccharides

Food is a complex matrix composed of numerous macromolecules composed of a sequence of monomers. These macromolecules are relevant in human nutrition by providing essential nutrients and influencing the inherent characteristics of foods and food‐based products. Among these diverse biomacromolecules, polysaccharides are dominant regarding abundance besides having a relevant impact on functional properties (Wang, Zhao, et al. 2023).

**FIGURE 1:**
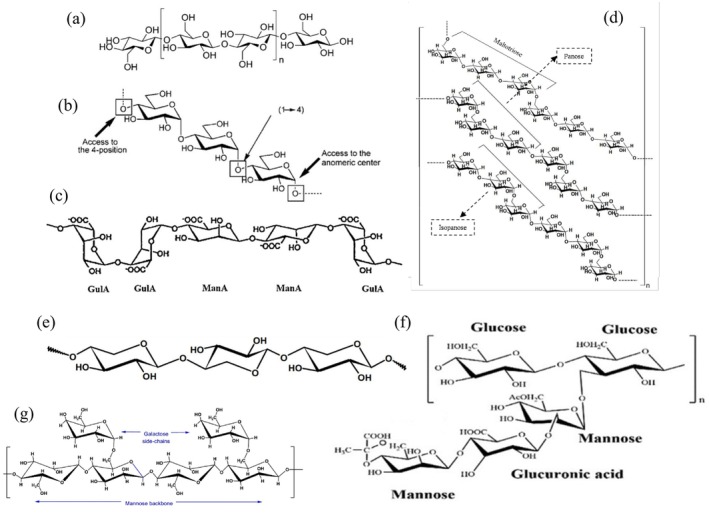
Chemical structure of some polysaccharides: (a) starch, (b) cellulose, (c) alginate, (d) pullulan, (e) xylan, (f) xanthan, and (g) guar.

Polysaccharides are large molecules made up of repeating monosaccharide units connected by glycosidic bonds. They are mainly composed of carbon, hydrogen, and oxygen, forming either linear or branched structures. The types of monosaccharides in polysaccharides vary; for instance, cellulose and starch are homopolysaccharides consisting solely of glucose, while hemicellulose and pectin are heteropolysaccharides that contain a variety of sugars like xylose, arabinose, galacturonic acid, and mannose (Figure 1a–g). The arrangement and type of linkages between these monosaccharides, such as α‐(1→4) or β‐(1→4) bonds in glucose‐based polysaccharides, play a crucial role in determining their structural and functional characteristics, which impact their solubility, digestibility, and potential uses in areas like food, pharmaceuticals, and bioengineering (Rostamabadi et al. 2023).

Dry grains are notably abundant in polysaccharides, mainly in the form of starch. In contrast, fruits and vegetables present a more varied polysaccharide composition, containing not only starch but also considerable amounts of fibers, pectins, and gums (Padil et al. 2020).

In food matrices, polysaccharides play key roles in providing structural support and serving as energy reserves. The plant cell walls and external tissues are mainly made up of cellulose and hemicellulose, which are crucial for maintaining structural integrity (Akeem Azeez and Olatunde 2018). These polysaccharides work in conjunction with other nutrients, such as proteins and lipids, to regulate the selective permeability of the cell wall and aid in the transport of molecules through diffusion processes. Starch, a major polysaccharide in plant matrices, serves as the primary energy storage form in plants, particularly in grains and cereals (Fernandes and Coimbra 2023; Nascimento et al. 2022; Hariprasad et al. 2024). Additionally, pectins and gums, such as apple pectin and beta‐glucans in oats, are present in food matrices (Moslemi et al. 2024; Yang, Cheng, et al. 2024). These compounds play a crucial role in maintaining cell turgidity, which refers to the internal pressure that contributes to the firmness of fruits.

In the food industry, polysaccharides unlock a wealth of functional properties. For instance, starch plays a pivotal role in baking, attributed to its distinctive thermal, pasting, structural, and chemical characteristics (Payling et al. 2020). Conversely, dietary fibers are increasingly recognized for their contributions to functional foods and products (Ragavan et al. 2022). Additionally, their properties make them highly advantageous in food packaging development, offering critical benefits such as biodegradability and enhanced resistance (Hariprasad et al. 2024).

To determine the quality and functionality of polysaccharides, evaluating their functional parameters is crucial. This evaluation is essential to achieving desired results in various food applications. A wide range of sophisticated technologies exists for this purpose, including spectroscopic, chromatographic, microscopic, and thermal analysis techniques (Kurzyna‐Szklarek et al. 2022; Wang, Zhao, et al. 2023).

### 2.1 Types of Food Polysaccharides

#### 2.1.1 Starch

Sugars combine to form polymers, with polysaccharides being complex carbohydrates made up of more than ten glucose units joined by glycosidic bonds (Rostamabadi et al. 2023). Starch is a prime example, where glycosidic bonds typically occur at the alpha (α) position between carbons 1 and 4 (α‐1,4) or between carbons 1 and 6 (α‐1,6) of glucose units. The location of these linkages determines the overall structure of the polysaccharide. When connected exclusively by α‐1,4 bonds, a linear chain known as amylose is formed. In contrast, the presence of additional α‐1,6 bonds leads to branching, resulting in a more intricate structure (Kishore et al. 2023).

The way these sugar units link together significantly impacts the properties of the resulting polysaccharide. Amylose forms a helical structure with only α‐1,4 linkages, while amylopectin, with its α‐1,6 branch points, has a more disorganized, amorphous structure (Kishore et al. 2023). The ratio of these structures in starch directly affects its behavior when heated (thermal properties), its ability to thicken solutions (pasting properties), its solubility in water, its susceptibility to breakdown by digestive enzymes, and ultimately, how it is used in various applications (Fonseca et al. 2019).

#### 2.1.2 Cellulose

Cellulose is a polysaccharide crucial for plant structure; it is composed entirely of β‐d‐glucose units linked exclusively by β‐1,4 bonds, resulting in a straight chain (Wang et al. 2024). Interestingly, its conformation allows individual cellulose chains to interact through hydrogen bonding and Van der Waals forces, forming microfibrils. These microfibrils aggregate into a robust fiber network, providing rigidity and protection for plant cell walls (Hariprasad et al. 2024).

Beta (β) linkages, particularly β‐1,4 linkages, promote the formation of straight, unbranched chains that can form rigid, fibrous structures due to extensive hydrogen bonding between individual chains. These β‐linked polysaccharides are typically insoluble in water and cannot be digested by human gut enzymes. However, they play a vital role in human health by regulating blood sugar levels, supporting the gut microbiota, and promoting regular bowel movements (Yang, Cheng, et al. 2024).

#### 2.1.3 Hemicellulose

Hemicellulose significantly influences the properties of food matrices due to its unique structure. Unlike linear, homogeneous cellulose, hemicellulose boasts a branched and highly variable composition (Arai et al. 2019). This polysaccharide is a diverse group of molecules, including xyloglucan, xylan, and glucomannan (Li et al. 2022). Its constituent sugars, primarily glucose, xylose, and glucose‐mannose sequences, are linked by β‐1,4 glycosidic bonds. Hemicellulose can have branched side chains containing xylose, galactose, arabinose, and carboxylic acids like glucuronic acid (Krawczyk et al. 2021). These distinctive structural features significantly impact how hemicellulose functions within food.

The branched structure and hydrogen bonding of hemicellulose contribute to a gel‐like network formation within the food. This network affects various textural properties, including water‐holding capacity and viscosity. Furthermore, the hemicellulose polymer's degree of polymerization, or chain length, can influence its susceptibility to enzymatic breakdown during digestion, impacting the bioavailability of nutrients within the food (Arai et al. 2019; Yang, Chen, et al. 2024).

#### 2.1.4 Chitin

Chitin, a polysaccharide second only to cellulose in natural abundance, is made of N‐acetyl‐D‐glucosamine units linked by specific β‐1,4 bonds. These linkages form crystalline microfibrils that provide structural support for the exoskeletons of arthropods and the cell walls of fungi and yeast. Commercially, chitin is extracted from crab and shrimp shells and is usually associated with proteins, pigments, and calcium carbonate. Notably, chitin exists in two primary forms, α and β, differentiated through spectroscopic and diffraction techniques (Rinaudo 2008). The α‐form—prevalent in lobsters, crabs, and insects—is the more abundant variant. However, chitin's insolubility in most common solvents presents a significant challenge for its processing and industrial utilization (Yang et al. 2020).

#### 2.1.5 Pectin

Pectin plays a vital protective role within the food matrix of fruits and tubers. This complex polysaccharide boasts a backbone of galacturonic acid residues linked by β‐1,4 glycosidic bonds. Decorating this backbone are various neutral sugars, such as rhamnose, arabinose, and galactose, alongside acidic sugars like glucuronic acid. These side chains, known as heterogalacturonans, can be highly branched and exhibit significant variability, even within a single fruit source (Hariprasad et al. 2024). This intricate structure is directly responsible for pectin's well‐known gelling and thickening properties, making it an essential ingredient in jams, jellies, and other food applications. Furthermore, pectin's ability to bind water significantly impacts the texture and shelf life of both plant‐based foods and processed food products (Kyomugasho et al. 2016).

Polysaccharides' properties are susceptible to improvement through chemical and physical processes. These modifications alter their structure, leading to functional changes (Rammak et al. 2021). Heat‐moisture treatment (HMT), extrusion, phosphorylation or acetylation esterification, and enzymatic treatments are some of the most common modification methods in starch (He et al. 2020). Also, microwave treatments can change starch properties by inducing complexation with other polymers (Frasson et al. 2024). As a result of these modifications, starches find applications as thickeners, gelling agents, moisture absorbers, stabilizers, and binders in various industries (Agyemang et al. 2020). Their wide range of functionalities, low cost, and low toxicity make starches highly valuable in the food, chemical, and pharmaceutical industries (Kochkina and Lukin 2020).

#### 2.1.6 Gums

Gums are a large group of carbohydrates consisting of multiple monosaccharide residues or their derivatives connected in a perplexing manner via a varied range of covalent, electrostatic, and hydrogen bonds. The term gum is usually used to distinguish those types of polysaccharides that do not participate in cell wall formation but are secreted in the form of exudates and are mostly pathological/physiological products. Given their outstanding attributes (i.e., biocompatibility, ease of accessibility, low cost, and non‐toxicity), gums have found a seat in various applications, including the production of tablets, the fabrication of biodegradable packaging materials (Mulla et al. 2023), the development of drug‐delivery systems with a sustained release, and acting as thickening/stabilizing components in food/pharmaceutical applications (Falsafi et al. 2023). The interest in broadening the applications of gums has given rise to their emergence in numerous additive manufacturing investigations as unique biopolymers with substantial potential for developing well‐structured 3D‐printed objects without the need to use synthetic polymers.

### 2.2 Recent Characterization Approaches of Polysaccharides

Polysaccharides, in addition to their vital functions in plant organisms, significantly influence both the technological and nutritional properties of various products (Nascimento et al. 2022). Owing to their renewable and eco‐friendly nature, low toxicity, and cost‐effectiveness, polysaccharides have been the focus of extensive research over the years (Hariprasad et al. 2024). These attributes make them highly suitable for diverse applications in the pharmaceutical and nutritional sectors, as well as a key ingredient in the food industry (Padil et al. 2020; Ragavan et al. 2022). However, given the wide variety of polysaccharides and the distinct organoleptic properties they impart, comprehensive evaluation remains essential (Rostamabadi et al. 2023).

Conventional methods for polysaccharide characterization, which integrate chemical and instrumental analyses, are often labor‐intensive, require extensive use of chemical reagents, and demand specialized expertise for operation. These drawbacks make them unsuitable for high‐throughput, automated, or real‐time measurements. Consequently, innovative approaches and advanced methodologies have been developed and refined to address these limitations.

#### 2.2.1 Spectroscopic Techniques

Polysaccharides are traditionally analyzed through colorimetric reactions, where the target group is separated and incubated with specific solvents, resulting in a colored solution. This solution is then analyzed using a spectrophotometer and quantified based on a standard curve. To minimize chemical usage, infrared (IR) spectroscopy has become a vital analytical tool, offering a more sustainable and efficient alternative for polysaccharide characterization.

The IR region of the electromagnetic spectrum is categorized into three subranges: near‐infrared (NIR) (12,800–4000 cm^−1^), mid‐infrared (MIR) (4000–400 cm^−1^), and far‐infrared (FIR) (950–750 cm^−1^). Among these, the MIR region provides critical insights into polysaccharides. This region is particularly useful for identifying functional groups such as CH, OH, NH, and SH, which are common components of plant‐derived organic molecules (Kurzyna‐Szklarek et al. 2022). Each functional group exhibits distinct characteristic bands, enabling precise identification. For carbohydrate analysis, the MIR spectrum is particularly valuable as it offers detailed information on the conformation (α or β) and ring structure (pyranoid or furanoid) of mono‐ and polysaccharides. Specifically, bands 2a (870–840 cm^−1^) and 2b (890 cm^−1^) are instrumental in distinguishing between α and β glucose conformers, with band 2a corresponding to the α conformation and band 2b to the β conformation (Oliveira‐Folador et al. 2018). Moreover, the particle size of the analyzed molecules significantly influences the spectral data, as ordered structures such as monosaccharides, disaccharides, and oligosaccharides produce narrow and sharp IR signals due to their well‐defined molecular arrangements.

Frasson et al. (2024) identified characteristic bands in modified avocado starches containing oil, indicating successful complexation, as these bands corresponded to those observed in the oil. Similarly, Kochkina and Lukin (2020) reported variations in the shape and intensity of peaks within a starch/chitosan/polyvinyl alcohol complex, depending on the ratios of the components used.

**FIGURE 2:**
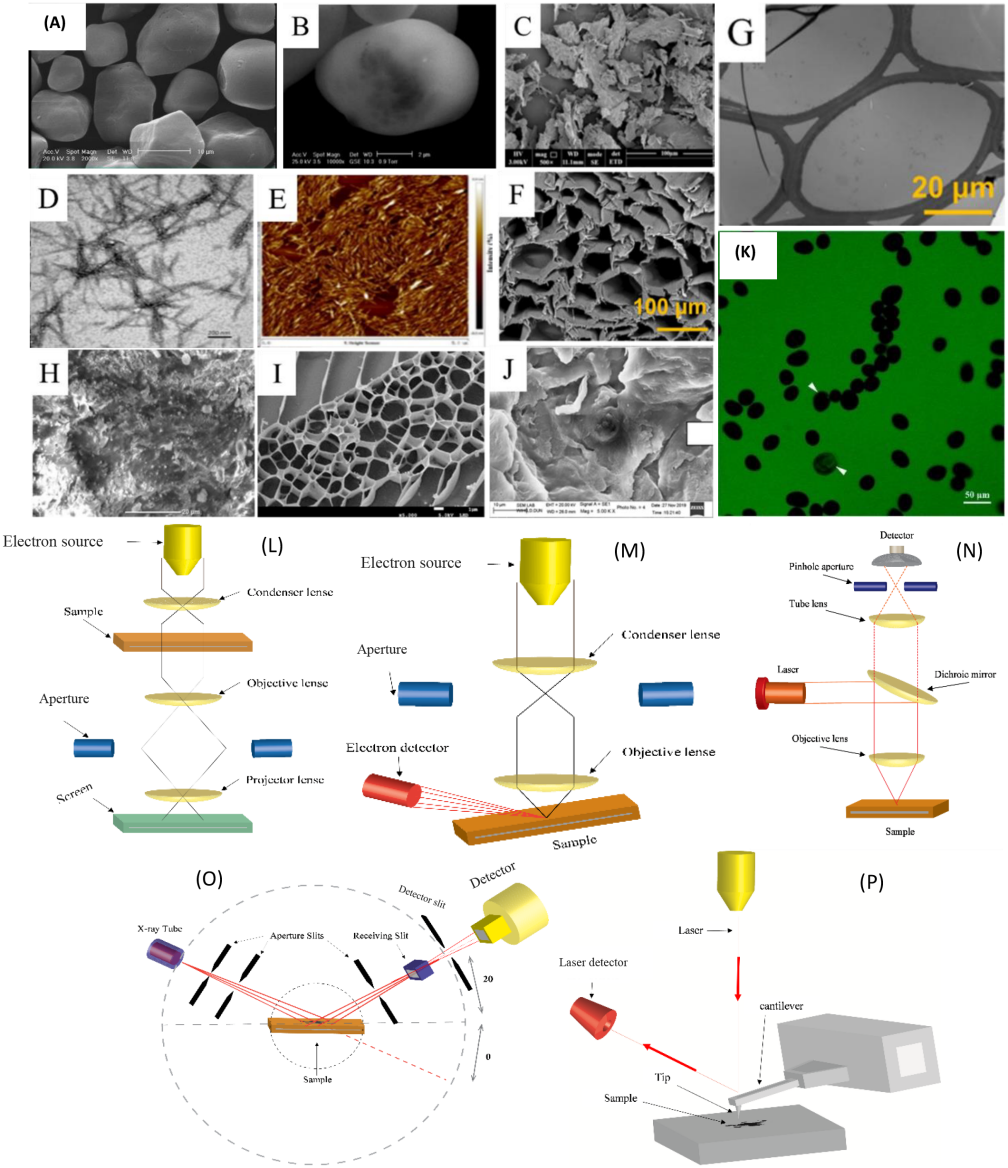
Polymers' microscopy using different approaches. SEM of native cornstarch (A) and ESEM micrographs of native wheat starch (B) from Barrera et al. (2013); SEM of black tea cellulose nanocrystals (C), TEM of black tea cellulose nanocrystals (D), and AFM of black tea cellulose nanocrystals (E) from Wang et al. (2024); SEM of hemicellulose from pristine bagasse cells (F) and TEM of hemicellulose from pristine bagasse cells (G), from Yang, Chen, et al. (2024); SEM of coffee pulp pectin (H), reprinted with permission from Biratu et al. (2024); SEM of cation‐facilitated pectin‐gel (I) from Kyomugasho et al. (2016); SEM of native guar gum (J) from Tyagi et al. (2020); CLSM images of potato starch (K), reprinted with permission from Chen et al. (2019); schematic diagram of instrumental setup of TEM (L), SEM (M), CLSM (N), XRD (O), and AFM (P).

X‐ray diffraction (XRD) utilizes a collimated X‐ray beam directed onto a polymer sample. As the X‐rays interact with the electron cloud surrounding the atoms in the polymer chains, they scatter, generating a detectable signal at specific angles (Figure 2O). Bragg's Law links the wavelength (*λ*) of the incident X‐rays, the interplanar spacing (*d*) within the polymer crystal, and the diffraction angle (*θ*). The arrangement of polymer chains plays a crucial role in determining the resulting diffraction pattern (Olawoye et al. 2022). Highly crystalline polymers, such as low‐amylose starches and cellulose, with well‐ordered chain packing, produce sharp, well‐defined diffraction patterns characterized by multiple distinct peaks. In contrast, amorphous polymers, like pectins, with more random chain conformations, generate broader and less distinct diffraction patterns (Frasson et al. 2024). Moslemi et al. (2024) observed no additional peaks in the XRD patterns of cobalt ferrite nanoparticles (CFNPs) incorporated into varying concentrations of apple pectin.

Solid‐state NMR has proven essential in revealing the structural role of pectin within plant cell walls. Pérez García et al. (2012) utilized advanced 2D and 3D magic‐angle‐spinning (MAS) SSNMR to analyze pectin branching and its influence on cell wall dynamics. Their findings showed that depectination alters intermolecular organization, leading to increased rigidity and denser polysaccharide packing. The suppression of pectin signals confirmed its removal, while enhanced cross‐peak intensities indicated its critical role in maintaining flexibility. By providing high‐resolution insights into pectin interactions, SSNMR underscores the significance of pectic polysaccharides in modulating the mobility and structural integrity of the plant cell wall. This study highlights the power of multidimensional NMR techniques in decoding complex biomolecular architectures (Pérez García et al. 2012).

#### 2.2.2 Chromatographic Techniques

High‐performance size exclusion chromatography with refractive index detection (HPSEC‐RI) separates molecules based on their hydrodynamic size. In this technique, the debranched polysaccharide sample is injected into a column packed with porous beads. Smaller molecules penetrate deeper into the pores, resulting in longer retention times, while larger molecules elute more quickly due to reduced interactions with the bead's internal surface. For instance, in the case of starch, this differential retention allows for the separation of amylose (smaller linear chains) from amylopectin (larger branched chains) within the debranched starch sample. A refractive index detector monitors the eluent as it exits the column, generating signals that correspond to the concentration of the eluting molecules. By analyzing the peak patterns and retention times, researchers can determine the relative quantities of molecules of various molecular sizes (Gonzalez and Wang 2023a).

High‐performance anion‐exchange chromatography with pulsed amperometric detection (HPAEC‐PAD) is an effective method for examining the complex structure of branched polysaccharides like amylopectin. This technique separates molecules based on their ionic interactions with a charged stationary phase in the column. The eluent, which contains a gradient of ionic strength, modulates these interactions. The debranched molecule is composed of linear chains of varying lengths (degree of polymerization), which elute at different times according to their ionic characteristics. The pulsed amperometric detector measures the electrical current generated by the analytes, offering a sensitive response for each eluting fraction. By analyzing the resulting peak profiles, researchers can categorize debranched fractions into distinct chain‐length groups. For instance, in amylopectin, they are categorized into A‐chains (shortest), B1‐chains, B2‐chains, and B3+ chains (longest). This detailed analysis provides a comprehensive understanding of the amylopectin architecture within debranched starch (Gonzalez and Wang 2023b).

Soyseven et al. (2022), using an HPLC technique, identified and quantified fructose, glucose, and sucrose monomers in 36 food samples, including fruits, using an isocratic acetonitrile‐water (78%:22%) with a 1.5 mL/min flow rate (Table 1). Santana Andrade et al. (2022) identified and quantified fructose, glucose, and sucrose monomers in cashew apples using an isocratic 0.5 mL/min water flow rate.

**TABLE 1:** Selected polysaccharides' characterization approaches.

Polysaccharide	Approach	Key findings	References
Starch	XRD	Characteristic peaks (A‐type) and a low damage degree were indicated by sharp peaks	Barrera et al. (2013)
	SEM	The granules presented a smooth and flat surface	
	ESEM	The granules presented smooth, flat granule surfaces and smooth edges	
	AFM	The granule surface looked (2D and 3D) smooth and polished, although some ridges appear to mark sites where granules were in close contact with each other	
Starch	DSC	Lower onset and peak temperatures than modified starches with autoclave and higher onset temperatures than microwave ones (M0% and M2%); higher Δ*H* values than autoclaved samples	Frasson et al. (2024)
	FTIR	Native and modified starches with 0% oil showed no difference in the wavenumber of the bands	
	XRD	Typical diffraction peaks of type A crystal structure are displayed around 2*θ* = 15°, 17°, 17.8°, 19°, and 23°	
Starch/chitosan films	XRD	Does not show any well‐defined peaks attributable to the crystal structures of these two biopolymers	Kochkina and Lukin (2020)
	FTIR	Some characteristic peaks reflecting the structure of both biopolymers are the broad band around 3270 and peaks located between 1632–1520 and 770–1120 cm^−1^	
	SEM	Homogeneous and compact structure without pronounced phase separation between these two biopolymers	
Cellulose, hemicellulose, and lignin	TGA	Decomposition of cellulose, hemicellulose, and lignin occurred between 200°C–300°C, 300°C–360°C, and 360°C–500°C, respectively	Wijaya et al. (2017)
Cellulose	FTIR	Characteristic peaks I crystal type, with some slight modifications indicating acid oxidation reactions	Wang et al. (2024)
	XRD	Typical diffraction peaks of type I cellulose are displayed around 2*θ* = 15.7°, 22.3°, and 34.6°	
	TG/DTG	The initial degradation temperature is at 220°C, and the weight loss rate reaches the maximum at 337°C	
Cellulose nanocrystals	SEM	Rod‐like morphology	Wijaya et al. (2017)
	XRD	Typical I crystal type, like the cellulose standard, with four distinct peaks at 2*θ* = 15.5°, 16.4°, 22.8°, and 35°	
	FTIR	Sharp and intense absorption band at 3427 cm^−1^ and similar stretching bands to cellulose I crystal type, but a new peak appears in the 1402 cm^−1^ spectrum, indicating the presence of sulfate esters	
	TGA/DTG	The initial degradation temperature was between 35°C and 200°C, and the weight loss rate reaches the maximum between 215°C and 353°C	
Hemicellulose	TEM	Some cracks on the surface	Yang, Chen, et al. (2024)
	XRD	Sharp characteristic peaks at (2*θ*), surface 200 and 101, were observed	
	HPLC	Confirmed that microwave treatment influences the extraction rate	
Hemicellulose	MALDI‐MS	Xylose was the main monosaccharide component, while glucose, arabinose, mannose, and galactose were present in low amounts	Arai et al. (2019)
Pectin	FTIR	Characteristic peaks at 3278, 2924, 2856, 1710, 1606, 1315, 1241, and 1020 cm^−1^	Biratu et al. (2024)
	SEM	Smoother and ruptures free surface	
	XRD	Sharp peaks at (2*θ*), indicating its crystallinity	
Pectin cobalt ferrite nanoparticles	DTA/TGA/DTG	The initial degradation temperature was at 380°C, related to residual water in the gel, and the weight loss rate reached the maximum between 380°C and 460°C	Moslemi et al. (2024)
	XRD	Peaks were identified around 2*θ* = 30.2°, 35.6°, 37.3°, 43.3°, 53.7°, 57.7°, and 62.9°	
	SEM	Spherical, with a few agglomerations and good dispersion	
	TEM	Cubic‐like particles with an average size of approximately 50 nm are agglomerated	
Pectin‐gel	SEM	Smaller and intact microstructures for low cations added samples	Kyomugasho et al. (2016)
Guar gum	TGA/DTA	The maximum weight loss occurred at 300°C	Tyagi et al. (2020)
	SEM	Irregular and rough surface	
Xyloglucan	MALDI‐MS	Three single xyloglucan oligosaccharide peaks with *m*/*z* values of 1085, 1247, and 1409 corresponded to the molecular masses of sodium adducts of XXXG, XXLG/XLXG, and XLLG where G, X, and L represent β‐d‐Glcp, α‐d‐Xylp‐(1,6)‐β‐d‐Glcp, and β‐d‐Galp‐(1,2)‐α‐d‐Xylp‐(1,6)‐β‐d‐Glcp, respectively	Wang et al. (2021)
36 food matrixes	HPLC‐ELSD	Quantified fructose, glucose, and sucrose from 36 food matrixes, including fruits, jams, and honey	Soyseven et al. (2022)

#### 2.2.3 Thermal Analysis Techniques

Differential scanning calorimetry (DSC) analysis is employed to evaluate the thermal properties of starch, specifically focusing on the gelatinization temperature range and the enthalpy of both gelatinization and retrogradation. This technique utilizes three key data points: onset temperature (*T*_*o*_), peak temperature (*T*_*p*_), and conclusion temperature (*T*_*c*_). By analyzing these points, it is possible to quantify the thermal energy required to disrupt the hydrogen bonds within the starch structure during gelatinization. In the context of starch thermal properties, *T*_*o*_ and *T*_*p*_ reflect the degree of order within the double helical structures and crystallinity of the starch granules, while the enthalpy change (Δ*H*) corresponds to the amount of these ordered helical structures present (Nascimento et al. 2024). Rostamabadi et al. (2023) mentioned in their review that ultrasound treatment employing 20, 25, and 28 kHz frequencies for 30 min at 25°C reduces the starches' thermal properties observed during DSC analysis.

#### 2.2.4 Fourier‐Transform Infrared Spectroscopy (FTIR)

Fourier‐transform infrared spectroscopy (FTIR) is a fundamental technique for unraveling the intricacies of polysaccharide structures due to its ability to reveal the specific functional groups that embellish these complex biomolecules. The obtained FTIR spectrum acts as a characteristic spectral signature, with peaks corresponding to the vibrational modes of various functional groups. These peaks provide crucial information about the monosaccharide constituents of the polysaccharide. For instance, the presence and intensity of OH stretching bands reveal the abundance of hydroxyl groups, a defining feature of all polysaccharides. Also, CH stretching vibrations can distinguish between different sugar units like glucose and mannose. The type of glycosidic linkages connecting these sugar units within the backbone (α or β) can be identified by analyzing specific peaks (Kurzyna‐Szklarek et al. 2022).

Furthermore, FTIR can detect modifications in the sugar units themselves. The presence of carbonyl groups (C〓O) indicates the presence of uronic acids in the polysaccharide, while sulfate ester groups (SO) are an indicative marker of sulfated polysaccharides. By analyzing the relative intensity of these peaks, researchers can estimate the extent of branching, acetylation, or sulfation in the sample. This information is invaluable in understanding the overall structure and properties of the polysaccharide. The ability to compare FTIR spectra of different polysaccharides allows researchers to differentiate between them based on their structural variations. It also helps understand how processing techniques or modifications might alter the structure of the polysaccharide (Saha and Roy 2022). While FTIR has limitations in determining the complete sequence of sugar units, it offers a rapid, non‐destructive approach for initial characterization due to the detailed functional groups' characteristic spectral signature.

Kochkina and Lukin (2020) observed methylene groups' (CH_2_) presence in the apple pectin's complex samples due to peaks at 2920 cm^−1^ and 2851 cm^−1^. Additionally, a peak at 1740 cm^−1^ suggests the presence of a carbonyl group (C〓O), which is likely retained from the acetate precursor during the synthesis of polyvinyl alcohol.

#### 2.2.5 Microscopic Techniques

The polymers' morphology is relevant to characterize the sample and confirm the processes' results and possible structural changes. Scanning electron microscopy (SEM) works with an electron beam generated using an electron source. This beam interacts with the atoms at the polymer surface, generating various signals. Detectors collect these emitted electrons, and the information obtained allows for a high‐resolution image of the polymer surface (Figure 2A,M). A conductive coating may be necessary to prevent charging induced by the electron beam and to work in a vacuum (Wijaya et al. 2017). Transmission electron microscopy (TEM) operates by transmitting a beam of electrons through a thin sample to generate an image or diffraction pattern, functioning under high‐vacuum conditions. This technique provides high‐resolution images at the nanometer scale, enabling the visualization of internal structures, including crystalline arrangements, phase boundaries, and defects, with exceptional detail (Figure 2D,L). Atomic force microscopy (AFM) operates by detecting cantilever deflections through the reflection of a laser beam, producing a topographic map of the sample surface. Unlike some other microscopy techniques, AFM does not require a vacuum, making it suitable for studying samples in various environments, including air, liquid, or under controlled conditions. This technique provides high‐resolution three‐dimensional images, revealing surface features such as texture, roughness, and nanostructures with remarkable precision (Figure 2E,P) (Wang et al. 2024). Environmental scanning electron microscopy (ESEM) operates on a similar principle to SEM but eliminates the need for dehydration and conductive coating. ESEM enables the direct examination of hydrated biological specimens in their native state (Barrera et al. 2013).

Padil et al. (2020) observed differences in SEM images at low and high magnification for uniaxial electrospinning in different gum‐based composite nanofibers fabricated. Wang et al. (2024) observed a dense distribution arranged in different directions, with a rod‐like structure, a large aspect ratio, and a certain degree of aggregation in black tea cellulose nanocrystals.

#### 2.2.6 Emerging Techniques

Advancements in technology drive the development of new methods and equipment. These advancements improve the resolution and accuracy of existing approaches and instruments.

In this category, confocal microscopy has emerged as a powerful imaging technique for studying macromolecular structures, enabling 3D reconstructions and capturing high‐resolution micrographs by utilizing a spatial filter to minimize beam aberrations (Figure 2K,N). A conventional CLSM setup consists of three primary components: a laser source, a detection system, and a scanning mechanism. During CLSM imaging, light emitted by the laser source passes through a pinhole aperture, forming an expanded parallel beam. This beam is directed to a dichroic filter, which deflects it by 90° toward the objective lens, focusing the laser onto a specific focal plane of the fluorescent sample. The scanning system then moves the laser beam systematically across the focal plane, pixel by pixel, along the *X* and *Y* axes. Fluorescent light reflected from the focal plane at each point is filtered through a pinhole to eliminate out‐of‐focus reflections that do not align with the focal point. Finally, a detector, such as a photomultiplier tube, captures the fluorescent signals from various sections of the focal plane.

Additionally, combining multiple techniques is highly recommended for comprehensive sample characterization. Wang, Zhao, et al. (2023) demonstrated that matrix‐assisted laser desorption/ionization (MALDI) is a powerful soft ionization technique for carbohydrate analysis. This technique complements mass spectrometers (MS) by enabling the determination of synthetic carbohydrates with high *m*/*z* values, reaching approximately *m*/*z* 24,600. MALDI‐MS generates relatively simple spectra, typically dominated by singly charged ions. Further fragmentation of these ions provides information on monosaccharide composition, linkage types, and sequence, allowing a rapid deduction of carbohydrate composition and structure (Wang et al. 2021). Compared to other MS technologies, MALDI‐MS offers significant advantages for determining the intact mass of high‐molecular‐weight polysaccharides.

## 3 Proteins

Proteins come in different shapes and sizes (molecular weights), with some being globular whereas others are fibrous, leading to diverse functions in nature. Proteins are complex macromolecules with important physical and functional roles in the organisms.

There are two recognized primary categories of simple proteins: globular proteins and fibrous proteins. Globular proteins are compact and generally have spherical or ovoid shapes. They play essential roles in living and dead organisms, serving as enzymes, hormones, and antibodies. Their large internal volume provides plenty of space for the creation of cavities with specific properties such as shape, charge, and hydrophobicity or hydrophilicity, which are crucial for substrate binding and catalysis (Rodwell and Murray 2018). Globular proteins can either dissolve or form colloidal suspensions in water and are relatively sensitive to changes in temperature and pH in comparison to the fibrous proteins. By contrast, fibrous proteins adopt extended conformations and serve as the primary structural materials in various biological tissues.

**FIGURE 3:**
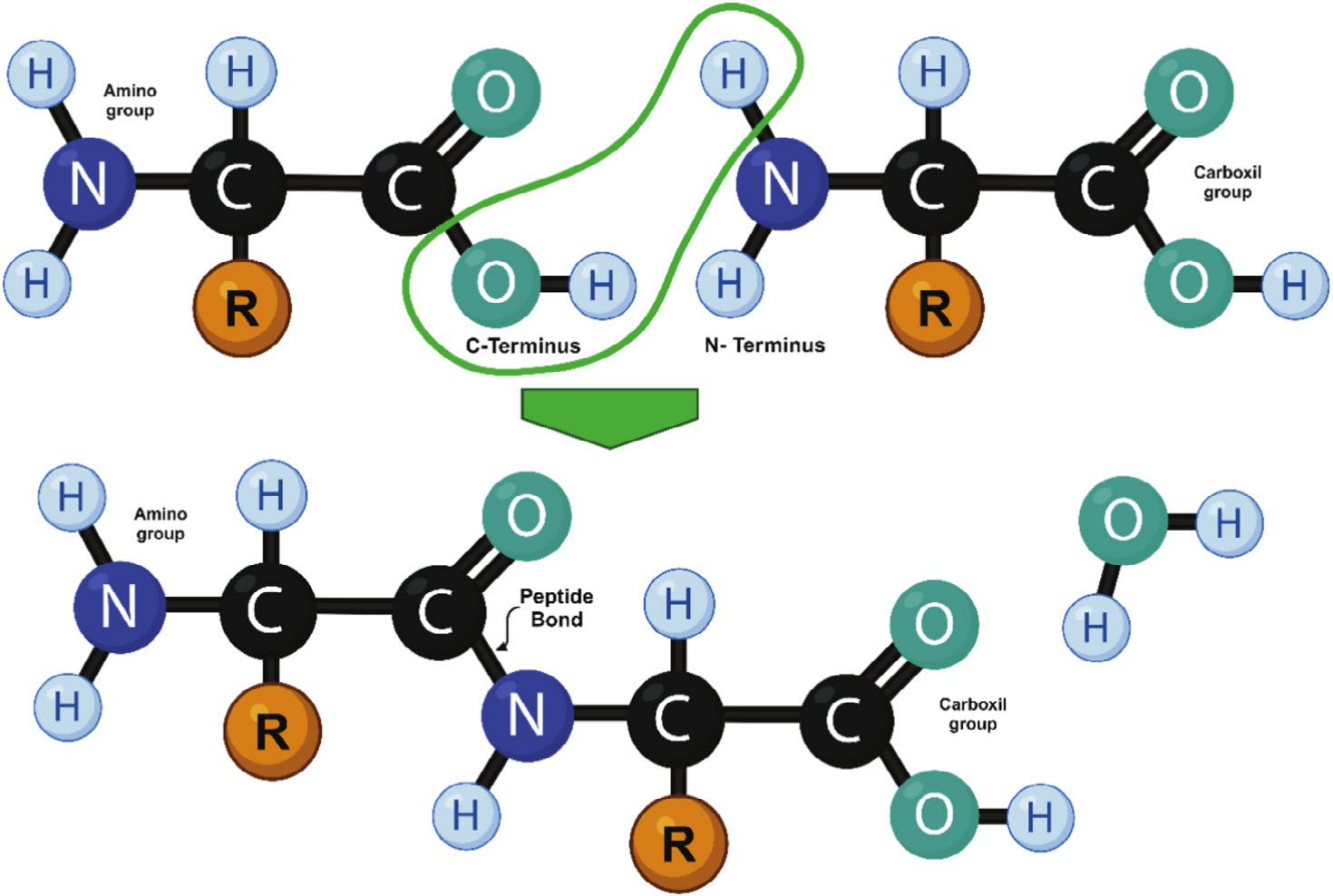
Formation of a peptide bond.

The essential building components of proteins are known as alpha amino acids (Rodwell and Murray 2018). There exists a total of 20 prevalent alpha amino acids commonly incorporated into the basis of protein structures across all life forms. The primary sequence of a protein is formed by linking the carboxylic acid of the upstream amino acid with the amine functional group to create an amide linkage known as the peptide bond (Figure 3). A chain of amino acids is known as a polypeptide. A protein's structure is upheld by noncovalent interactions that are individually weaker than covalent bonds. These noncovalent interactions include hydrogen bonds (between polar groups), ionic interactions (between charged groups), hydrophobic interactions (among nonpolar groups in aqueous solution), and van der Waals interactions, all having substantially lower energy than covalent bonds. The large number of weak interactions within supramolecular complexes stabilize these assemblies, producing their distinctive structures (Nelson and Cox 2017).

### 3.1 Protein Shape and Levels of Structure

Proteins are macromolecules composed of basic units called amino acids, which contain carbon, hydrogen, oxygen, nitrogen, and sulfur. Protein molecules are constructed from one or more twisted and folded strands of amino acids (Jakubowski and Flatt 2024).

**FIGURE 4:**
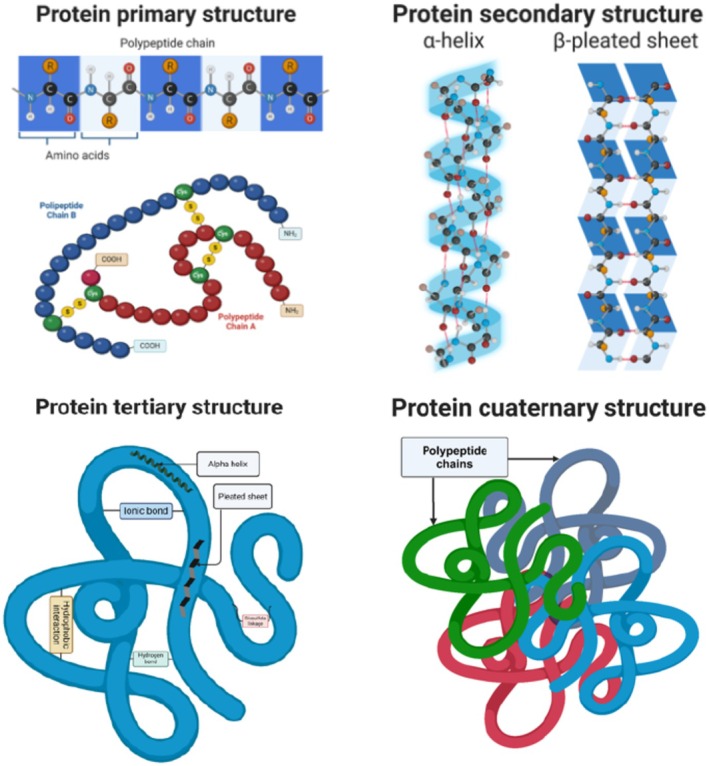
The four different aspects of a protein structure: Primary, alpha‐ helix and beta‐pleated sheets, tertiary, and quaternary protein structure.

Most proteins fold into unique three‐dimensional structures. The shape that a protein folds into naturally is known as its native conformation. While most proteins can fold unassisted through the chemical properties of their amino acids, others require the aid of molecular chaperones (ATA 2020). There are four distinct aspects of a protein's structure; primary structure, where a sequence of the amino acids is in a polypeptide chain; secondary structure, where the folding of short (3‐ to 30‐residue) contiguous segments of polypeptide are into geometrically ordered units; Tertiary structure, where the three‐dimensional assembly of secondary structural units form larger functional units such as the mature polypeptide and its component domains; and quaternary structure, where the number and arrangement of polypeptide units form oligomeric proteins (Rodwell and Murray 2018) (Figure 4). The characterization of these levels is useful in comprehending the structure and physical–chemical and biological properties of the proteins. The four levels also interdependently influence a protein's biological function. The first three structural levels can exist in molecules formed of a single polypeptide chain, whereas the fourth involves multi‐chained protein molecules (Kurunczi and Oprea 2003). Each level of structure can be used to identify a protein. Every protein contains at least a primary, secondary, and tertiary structure. While only some proteins have a quaternary structure (Sanvictores and Farci 2022).

### 3.2 Protein Analysis Techniques

Proteins differ among them in type, number, and sequence of amino acids making up the polypeptide backbone (Guo et al. 2025). Therefore, they have different physicochemical properties, molecular structures, and nutritional attributes. There is a wide range of experimental techniques used for the detection, purification, identification, and characterization of their structure and to determine the function of proteins. Protein analysis methods may also involve the analysis of the entire protein complement of a cell, tissue, or organism under specific conditions. Thus, various methods are currently used to identify and verify protein interactions and to quantify protein expression levels. Currently, there are three major techniques for protein analysis, that is, protein separation, protein identification, and Western blotting (Stone 2022).

#### 3.2.1 Protein Separation

Protein electrophoresis allows proteins to be separated or purified by placing them in a gel matrix in the presence of an electric field, allowing observation of the mobility of the proteins. The most widely used technique for protein separation is sodium dodecyl sulfate polyacrylamide gel electrophoresis (SDS‐PAGE). This technique, widely used in biochemistry, genetics, and molecular biology, allows proteins to be separated based on their size, solubility, charge, and binding affinity. Hence, SDS‐PAGE separates proteins primarily based on molecular weight (ATA 2020).

Other methods include:

##### 3.2.1.1 Isoelectric Focusing

In this technique, different molecules are separated by their differences in electrical charge. This is usually performed in gel and takes advantage of the change of charge of a molecule in a different environmental pH.

##### 3.2.1.2 Chromatic Methods

There are two chromatographic methods frequently used for protein separation: high‐performance liquid chromatography (HPLC) and thin‐layer chromatography (TLC). Both are complements to gel‐based methods. Although chromatography is a common technique in biochemistry laboratories used for the purification, identification, and quantification of protein mixtures, laser diffraction is traditionally used for precolumn sizing and polydispersity management.

##### 3.2.1.3 Two‐Dimensional Gel Electrophoresis

This is a powerful gel‐based method that is commonly used to analyze complex samples in order to characterize the full range of proteins in the sample, not just a few specific proteins.

#### 3.2.2 Western Blot

Immunoassays such as Western blotting and enzyme‐linked immunosorbent assay (ELISA) are well‐established protein detection and quantification techniques (Xiong et al. 2022). The Western blotting technique uses three steps to identify specific proteins from a complex mixture of proteins: separation by size, transfer to a solid support, and labeling of the target protein using a primary and secondary antibody suitable for visualization. Besides, immunoblotting is the most used version of the Western blot technique. This technique is used to detect specific proteins within a given tissue homogenate sample. The protein sample is first electrophoresed by SDS‐PAGE to separate the proteins based on molecular weight. The proteins are then transferred to a membrane where they are probed using antibodies specific for the target protein.

At present, there is a sensitive, luminescence‐based, and homogenous immunoassay alternative. This technique offers benefits emphasized by real‐lab data for the detection of cytokines, post‐translational modifications (PTMs), and antibody binding.

### 3.3 Protein Identification

There are two methods commonly used to identify proteins: Edman degradation and MS. Developed by Pehr Edman, Edman degradation was developed for sequencing amino acids into a peptide. Here, the amino‐terminal residue is labeled and cleaved from the peptide without modifying the peptide bonds between other residues of amino acids.

MS is an analytical technique used for protein analysis. It determines the masses of particles by measuring the mass‐to‐charge ratio of charged particles. It also studies the elemental composition of a molecular sample and the peptides chemical structure. In fact, MS is a leading analytical method in proteomics (Mann et al. 2001). MS is used for protein identification, the study of protein–protein interactions, characterization of PTMs, and protein quantification. For these applications, the proteins of interest have to be digested with an enzyme such as trypsin, and the resulting peptides are analyzed by MS. Trypsin cleaves proteins into peptides with an average size of 700 to 1500 Da, which is in the ideal range for MS (Laskay et al. 2013). The identification of peptides and, subsequently, proteins is completed by matching the ion spectra of peptide fragments with theoretical spectra generated from protein databases. Identification can be done through:

- Peptide fingerprinting: This method uses the masses of proteolytic peptides as input to a search of a database of masses from the digestion of a list of known proteins. This method is not dependent on protein sequencing for protein identification, this being an advantage. However, it requires that the database contain the protein that is already characterized in another organism.
- De novo peptide sequencing: This method can obtain peptide sequences without a protein database and uses computational techniques to deduce the peptide sequence from experimental tandem mass spectrometry (MS/MS) spectra. It can be used for antibodies, unsequenced organisms, peptides with PTMs, and endogenous peptides.

#### 3.3.1 Considerations of Each Method

Immunoassays are suitable for confirming the presence of specific proteins, quantification of proteins, and identifying the presence and/or extent of PTMs. On the contrary, MS is ideal for characterizing proteins and protein complexes. MS identifies and quantifies specific PTMs with high sensitivity and resolution. Zhang et al. (2022) established a precise method for quantifying and identifying bovine IgG subtypes using high‐resolution mass spectrometry (HRMS) and targeted proteomics. Four peptides were selected based on specificity, enzymatic hydrolysis, and stability, with isotope‐labeled peptides generated via isotope dimethylation serving as internal standards. Ultrahigh‐performance liquid chromatography–mass spectrometry (UHPLC–MS) facilitated IgG detection in milk, and HRMS confirmed characteristic peptides qualitatively, validating the method's effectiveness (Zhang et al. 2022). Nevertheless, MS is not suitable to identify rare proteins in a complex sample. New techniques such as next‐generation sequencing (NGS) can excel at detecting modifications that might remain obscured in MS or unidentified in immunoassays (Moretti 2024).

### 3.4 Modern Protein Analysis Techniques

The analysis of protein complexes involves a comprehensive interpretation of the structure and function of proteins, which are present in complex biological samples. Modern techniques allow us to study proteins more effectively and efficiently, reducing costs. They include:

#### 3.4.1 Light Scattering

Any increase in the size of a protein will likely be the result of the formation of aggregates. The sensitivity of the light scattering measurement to larger proteins will result in changes in the mean hydrodynamic size due to denaturation.

##### 3.4.1.1 Batch Dynamic Light Scattering (DLS)

**FIGURE 5:**
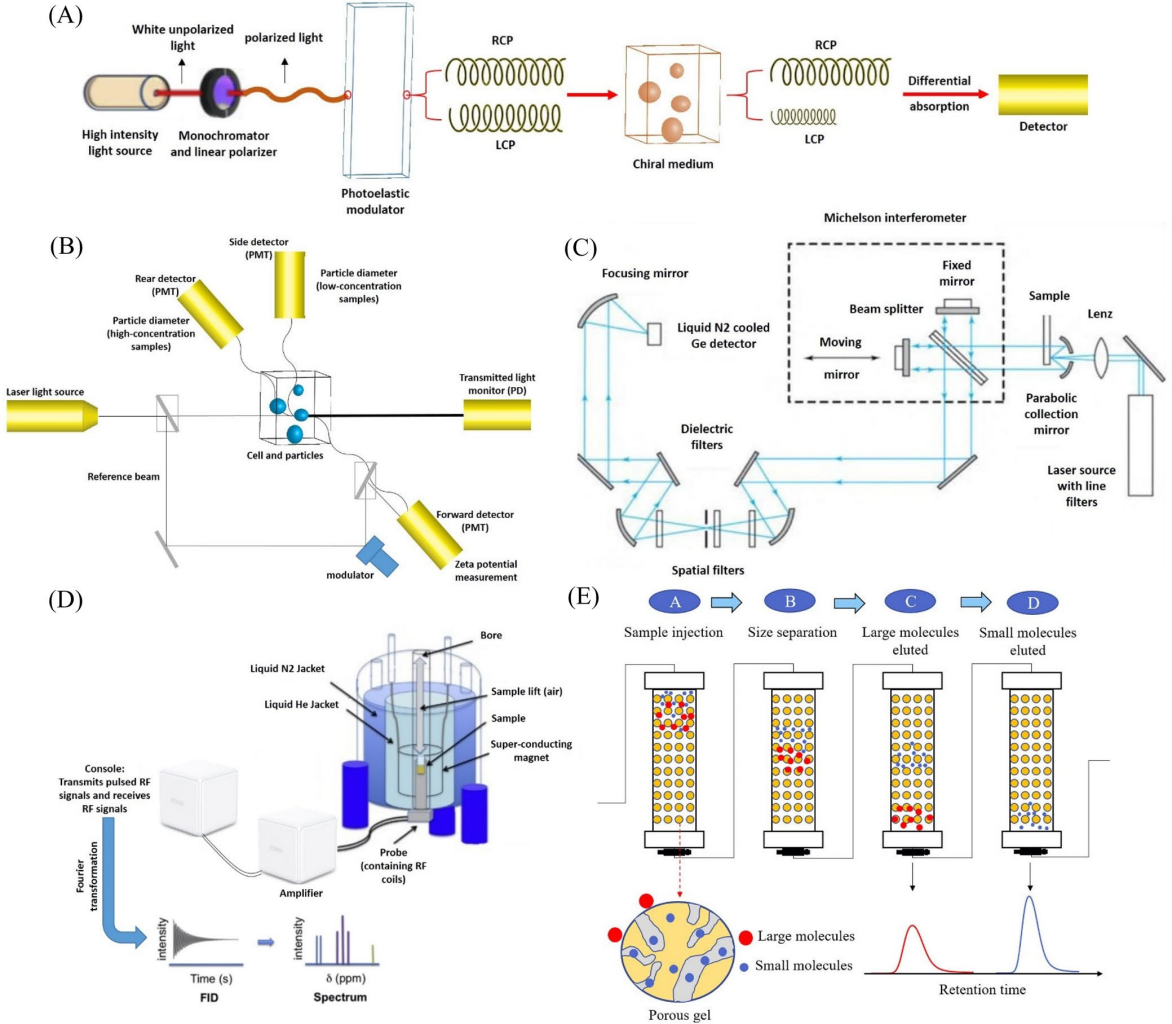
Schematic illustration of different characterization instruments: (A) Circular dichroism, reprinted with permission from Rostamabadi et al. (2020); (B) Dynamic light scattering, reprinted with permission from Choudhary et al. (2017); (C) FITR, reprinted with permission from Vallikkodi (2018); (D) NMR, reprinted with permission from Rankin et al. (2014); (E) Gel‐permeation chromatography, reprinted with permission from Ma et al. (2021).

Since proteins have a very consistent composition and fold into tight structures, the hydrodynamic size relates predictably to molecular weight (Figure 5B). Size can also be used as a predictor of activity. DLS is positioned as the most sensitive technique for detecting small quantities of aggregates in protein preparations.

##### 3.4.1.2 Static Light Scattering (SLS)

Following on from DLS measurements, SLS measurements can also be made on proteins. The molecular weight, which is proportional to the amount of light scattered, can be calculated by measuring the amount of light scattered at different concentrations of a sample. This technique can also be useful for studying crystallization conditions. A strongly positive value indicates good solubility, while a strongly negative value indicates a tendency to aggregate.

##### 3.4.1.3 Charge and Zeta Potential

A significant number of the functional groups on amino acids can be charged, and any combination of these may be in their charged or uncharged states in the protein. This will change depending on the conditions in the local environment. Overall, zeta potential is a measurement of the strength of repulsive forces among molecules in a solution. Conventionally, this has been used as a primary indicator of the stability of a sample preparation. With high zeta potential, a protein preparation can be expected to be stable for longer periods than a similar preparation with a low zeta potential.

#### 3.4.2 Multi‐Detection GPC/SEC

Gel‐permeation/size exclusion chromatography (GPC/SEC) is the ideal technique for characterizing macromolecules such as proteins (Figure 5E). The information offered from GPC/SEC analysis is very vast; it includes molecular weight (MW), molecular size in the form of hydrodynamic radius (Rh) and radius of gyration (Rg), intrinsic viscosity (IV), concentration, compositional analysis, and branching data, among other parameters. While DLS can be used to characterize the oligomeric state of a protein, it is unable to resolve a mixture of oligomers. However, GPC/SEC separates molecules based on their size, making it an excellent partner for light scattering; in this way, the different components in a mixture are identified.

#### 3.4.3 Circular Dichroism Spectrometry

Circular dichroism (CD) is an absorption spectroscopy method based on the differential absorption of left and right circularly polarized light (Figure 5A). Optically active chiral molecules will preferentially absorb one direction of the circularly polarized light. The difference in absorption of the left and right circularly polarized light can be measured and quantified by this technique. Some spectrometers provide maximum signal‐to‐noise under high‐absorbing, low‐light‐intensity conditions of the far‐UV spectral region to explore the structure and stability of biomolecules.

#### 3.4.4 Isothermal Titration Calorimetry

Isothermal titration calorimetry is a physical technique used to determine the thermodynamic parameters of interactions in a solution. It is most often used to study the binding of small molecules to larger macromolecules (proteins, DNA, etc.).

### 3.5 Other Protein Analysis

Crystallization of proteins is a necessary step for elucidating their detailed three‐dimensional structure, and it requires a highly purified protein kept in ideal conditions. In crystallization, the polydispersity is a measure of the purity of a sample. A protein that is highly purified has very low dispersity, and it also indicates that all the protein is in one particular oligomeric conformation and its structure is very well controlled. All of which are required for crystallization. Once a protein sample with the lowest polydispersity is identified, it is possible to find the most suitable conditions for crystallization.

The size and quaternary structure of the protein can also be used as a predictor of activity to be studied. Many proteins rely on correct quaternary structure in order to function, so again, hydrodynamic size can be used as a predictor of activity. When proteins oligomerize, their size and molecular weight will increase. By measuring the protein under different conditions, the oligomeric state of the protein can be assessed.

#### 3.5.1 Bioluminescent Technologies

There are bioluminescent technologies for studying protein interactions in live cells. They are applied to investigate kinase biology. Cellular processes such as signaling, division, growth, development, differentiation, and death in cells are orchestrated by kinases. The disruption of kinases in intracellular signaling networks leads to diverse diseases, including cancer. Consequently, kinases have become an important target for drug discovery. The interaction of bromodomain proteins with histones is of current high interest for drug targeting, as modulation of this interaction has been implicated in disease. Some methods monitor intracellular and real‐time inhibition of binding to chromatin after treatment of cells with inhibitors. These techniques can measure intracellular protein–protein interactions in living cells, use full‐length proteins, and monitor interacting pairs closer to physiologically relevant protein levels (Promega 2024).

#### 3.5.2 Next‐Generation Sequencing (NGS)

While commonly associated with DNA and RNA sequencing, NGS has extended its applications to protein sequencing, improving the speed and ease with which researchers can correlate biological function with changes in protein sequence and modifications. NGS can facilitate de novo protein sequencing, a method in which the amino acid sequence of a protein is determined directly without prior knowledge of its DNA. This is useful for discovering new proteins or protein variations.

New NGS platforms enable very sensitive experiments and allow researchers to study proteins that are in lower abundance. These advanced technologies offered novel and rapid ways to sequence static genomes as well as entire transcriptomes for expression analysis under different conditions. New technologies are evolving rapidly, and addressing prospective issues like the amelioration of protocols for generating sequencing libraries, offering new strategies for data analysis, and, most importantly, restructuring and revamping experimental design (Katara et al. 2024).

#### 3.5.3 Cryo‐Electron Microscopy (Cryo‐EM)

Cryo‐electron microscopy (cryo‐EM) is a structural biology technique used to determine the 3D structures of biomacromolecules, particularly proteins and protein complexes, in food science. Unlike X‐ray crystallography, cryo‐EM enables structural characterization without crystallization, preserving proteins in their native states by freezing hydrated samples and capturing high‐resolution images via TEM. Cryo‐EM is valuable for analyzing protein–protein interactions, conformational changes, and structural heterogeneity in food proteins, offering insights into functional states, stability, and molecular mechanisms of enzymes and structural proteins in food processing. Its ability to study dynamic and heterogeneous protein complexes makes it superior to traditional methods. Advancements in image processing algorithms and direct electron detection have enhanced cryo‐EM resolution, enabling near‐atomic structural determination of food proteins. The technique is increasingly used in food quality assessment, allergen detection, and nutritional profiling, supporting the development of functional food ingredients and improved formulations (Nogales and Scheres 2015).

### 3.6 Analysis of Amino Acids

Protein amino acid analysis is a technique for determining the type and amounts of amino acids in a protein sample, providing information on protein composition, PTMs, and overall protein quality. By gaining insight into amino acid content, researchers can assess protein purity, determine changes in amino acid composition, and evaluate protein stability.

The main techniques for protein amino acid analysis are ion exchange chromatography, high‐performance liquid chromatography, gas chromatography (GC), and capillary electrophoresis. The instruments used in protein amino acid analysis include amino acid analyzers, UV–Vis spectrophotometers, and mass spectrometers.

The process of protein amino acid analysis in creative proteomics involved the following sequence: sample preparation, hydrolysis, derivatization, LC‐separation, MS detection, and data analysis. The applications of protein amino acid analysis include assessing protein quality, validating protein structure, studying PTMs, and assessing protein purity (Satinder and Scypinski 2001; Kambhampati et al. 2019).

A study by Liu et al. (2025) employed multiple advanced techniques to characterize soy peptides and their anti‐inflammatory effects. Simulated digestion and absorption revealed peptide transport efficiency, highlighting the role of N‐terminal residues. MS provided detailed sequence analysis, identifying key amino acid patterns linked to function. Molecular docking predicted peptide interactions with IκB, confirming their stabilizing potential. Cellular assays validated these findings, demonstrating reduced inflammatory cytokine levels. Together, these methods offer crucial insights into peptide bioactivity, advancing protein characterization and functional food development (Liu et al. 2025).

### 3.7 Characterizing Protein–Macromolecule Interactions

Protein–carbohydrate interactions are essential in food science, influencing texture, stability, and functionality in processes like dough formation, emulsification, and gelation. Lectins, glycoproteins, and enzymes mediate these interactions, governed by hydrogen bonding, hydrophobic forces, and electrostatic mechanisms, impacting food quality and bioavailability. Maillard reactions and protein‐polysaccharide complexes influence characterization by inducing structural changes detectable through SDS‐PAGE, circular dichroism spectroscopy, and SEM. Glycosylation leads to new molecular weight bands, confirming successful conjugation, while secondary structure alterations increase random coil content measurable via CD spectroscopy. However, aggregation, cross‐linking, and molecular weight shifts complicate precise molecular identification, particularly in MS and chromatography‐based techniques. Despite advancements in XRD, NMR, and cryo‐EM, determining protein‐carbohydrate complex structures remains challenging, requiring improved refinement protocols. NMR studies face low sensitivity, mitigated by higher magnetic fields. Computational methods, particularly molecular docking, predict complex structures, with Vina‐Carb being the most effective carbohydrate docking program. However, its inability to account for CH–*π* interactions limits its accuracy in predicting carbohydrate binding to aromatic amino acids (Zhang et al. 2022).

A study by Tao et al. (2024) outlines several common pretreatment and extraction methods used to optimize glycosylation and improve characterization of soybean protein isolate (SPI) and soybean peptide (SP) with ginseng polysaccharide (GP). The study employed alkali extraction‐acid precipitation to prepare protein‐polysaccharide mixtures before glycosylation, ensuring effective conjugation. The reaction conditions were carefully controlled by adjusting the pH to 10 and heating the SPI/SP solutions in a water bath at 45°C for specific time intervals (25–65 min) to facilitate Maillard reaction‐induced glycosylation. Following glycosylation, samples were freeze‐dried to improve stability and prevent degradation. The study also utilized SDS‐PAGE electrophoresis to analyze molecular weight changes in the conjugates and circular dichroism spectroscopy to assess alterations in secondary structure. Additionally, SEM was employed to observe micromorphological modifications resulting from protein unfolding and glycosylation. These methods collectively enhanced the characterization of glycosylation products while addressing potential challenges posed by macromolecular interactions (Tao et al. 2024).

### 3.8 Emerging Techniques

A dual signal light detection method based on porous silicon Bragg mirror (PSBM) and quantum dot (QD) labeling was developed by Gao et al. (2022) for β‐lactoglobulin (β‐lg) detection. The first signal light, a 633 nm laser probe, was reflected from the PSBM surface, positioned at the band gap edge for minimal reflectivity. β‐lg antibodies were labeled with CdSe/ZnS QDs and reacted with β‐lg molecules fixed to the inner walls of porous silicon pores. Specific biomolecular binding in PSBM increased the refractive index, enhancing reflected light detection, with QDs amplifying refractive index changes. The second signal light, QD fluorescence at 630 nm, was excited by a short‐wavelength laser and further enhanced by PSBM. Superimposed images of both signals were captured simultaneously using a digital microscope, and β‐lg detection was achieved by analyzing the average gray value change before and after the biological reaction. The detection limit was 0.12 ng/mL, demonstrating the method's effectiveness in identifying cow milk adulteration in β‐lg‐free camel milk (Gao et al. 2022).

Targeted proteomics enables precise quantification of proteins based on peptide‐protein quantum transfer relationships, with characteristic peptides selected for specificity, sensitivity, and stability. Recent advancements in artificial intelligence (AI) have enhanced protein characterization by enabling predictive modeling and high‐throughput data analysis. AI‐driven techniques, such as deep learning‐based protein structure prediction (e.g., AlphaFold2), facilitate accurate modeling of protein complexes, aiding in food formulation and ingredient optimization. AI also supports pattern recognition in MS data, improving the identification of bioactive peptides and allergens in food products. These methods collectively contribute to food quality assessment, allergen detection, nutritional profiling, and functional ingredient development, ensuring the safety and efficacy of food products (Wodak et al. 2023).

## 4 Food Lipids

Lipids are one of the major components of both plant‐ and animal‐derived food. Lipids play a crucial role in our body since they are essential components of the cell membrane structure, function as a storage form of energy, and serve as a metabolic fuel. Certain fatty acids (FAs) and fat‐soluble vitamins are essential nutrients because our body is unable to synthesize them, while others primarily function as a source of energy. Lipids are substances that have low solubility in water but can dissolve in nonpolar solvents like diethyl ether, petroleum ether, benzene, chloroform, hexane, etc. (Shahidi et al. 2017; Dhara and Nayak 2022). Given the complexity and variability of lipids, it is challenging to provide a precise definition due to varying perspectives. Numerous categorization schemes have been suggested for lipids. From a nutritional perspective, as stated in the National Academy of Sciences study on nutrition labeling, oils and fats are classified as complex organic compounds resulting from the combination of three FA molecules with one molecule of glycerol (Shahidi et al. 2017). The U.S. Food and Drug Administration (FDA) has traditionally defined total fat of food as “the sum of the components with lipid characteristics that are extracted by Association of Official Analytical Chemists (AOAC) methods or by reliable and appropriate procedures.” The FDA has changed from a definition based on solubility to a new definition of “total lipid FAs expressed as triglycerides”, in order to accurately quantify caloric FAs (O'Keefe and Sarnoski 2017).

Lipids can be classified according to their physical characteristics at room temperature, with fats being in solid form and oils being in liquid form. They can also be classified based on their polarity, with some lipids being polar and others being neutral. Furthermore, lipids can be categorized as essential or nonessential FAs depending on their importance for human health. Lastly, lipids can be categorized as simple, complex, or derived based on their structural composition. The classification based on structure is preferred among all classifications. This is because the classification of different polarity groups does not involve certain lipids, such as short‐chain FAs, within the appropriate category. Additionally, the other two classification systems are highly uncertain and insufficiently specific for these compounds (O'Keefe and Sarnoski 2017). The structural classification of lipids is based on the number of products generated through hydrolysis. Simple lipids (homolipids) yield a maximum of two types of structural moieties, primarily FAs and alcohol components (e.g., triacylglycerols, cholesteryl esters, waxes, ceramides, etc.). On the other hand, complex lipids (conjugated lipids) produce more than two types of moieties through hydrolysis (e.g., glycerophospholipids (phospholipids), glyceroglycolipids (glycolipids), and sphingolipids). Simple lipids consist of esters formed from FAs and alcohols, whereas complex lipids are esters of FAs and alcohol that also contain other groups such as amine derivatives or amino acids, phosphorus, protein, or carbohydrates. Derived lipids are produced or released from simple or complex lipids by hydrolysis (e.g., FAs, fatty alcohols, fat‐soluble vitamins, sterols, hydrocarbons, etc.) (Asokapandian et al. 2021; Domínguez et al. 2022; Shahidi et al. 2017; Fahy et al. 2005; O'Keefe and Sarnoski 2017). All or any of these lipid molecules can be found in food, but the most prevalent and significant ones are triacylglycerols (TAGs) and phospholipids (PLs). Solid TAGs are referred to as “fats,” which are typically derived from animals (e.g., lard and tallow), while liquid TAGs are referred to as “oils,” which are generally of plant or marine origin (e.g., vegetable and marine oils) at room temperature. Fats and oils show variations in texture, functionality, and appearance because of the differences in the types of FA chains that bond to form triglycerides (Asokapandian et al. 2021; Shahidi et al. 2017; Srigley and Mossoba 2017). FAs are the primary constituents of fats and oils. FAs are organic acids that are composed of a long‐chain, straight hydrocarbon with at least one carboxyl group. FAs usually are obtained from triglycerides and phospholipids.

The fatty acid may be represented by the common formula R–COOH, where R denotes a hydrocarbon chain (Asokapandian et al. 2021). FAs can be classified according to the degree of unsaturation (number of double bonds) as either unsaturated fatty acids (UFAs) or saturated fatty acids. Saturated fatty acids (SFAs) are characterized by the absence of double bonds and complete saturation with hydrogen atoms. Saturated fats have a greater melting point and show lower reactivity compared to unsaturated fats. Palm oil, coconut oil, butter, and tallow are among the sources of SFAs. UFAs, predominantly included in plant oils, nuts, seeds, and fish, provide a multitude of health advantages. Their beneficial effects extend to cardiovascular health, metabolic function, immunological response, and even mental well‐being. UFAs can be subdivided into two categories: monounsaturated (MUFA), which have one unsaturation, and polyunsaturated (PUFA), which have two or more unsaturations. Furthermore, PUFA can be divided into two different groups known as omega‐6 (linoleic acid) and omega‐3 (linolenic acid). These groups differ in the position of the unsaturation of the double bond, their chemical composition, the position of the unsaturation of the double bond, as well as their natural existence and biological roles (Asokapandian et al. 2021; Domínguez et al. 2022). Linolenic acid serves as a precursor to eicosapentaenoic acid (EPA) and docosahexaenoic acid (DHA). It plays a vital role in important biological processes, such as regulating inflammatory and immunological responses (Cardoso et al. 2021). The chemical and physical properties of FAs, such as solubility and melting point, are affected by the number of carbons and the degree of unsaturation. Furthermore, it is important to acknowledge that the proneness of FAs to oxidative processes increases as the degree of unsaturation increases, along with the variations in their physical properties (Asokapandian et al. 2021; Domínguez et al. 2022). Autoxidation (lipid oxidation) is a chemical reaction that occurs when molecular oxygen reacts with UFAs and is initiated by free radicals. The oxygen‐dependent deterioration of lipids has been widely acknowledged as an important problem in the preservation of fats and oils. Lipid oxidation products cause undesirable flavors and aromas and reduce the nutritional value and safety of fats and oils, resulting in the formation of toxic compounds. The oxidation process in fats and oils involves the degradation of lipids that contain UFAs like linoleic, linolenic, oleic, and long‐chain PUFAs. During this process, however, other unsaturated lipids found in fats and oils, such as cholesterol and other sterols, also undergo oxidation (Miraliakbari and Shahidi 2008; Miyashita et al. 2018). When studying the impact of UFAs on nutrition, it is important to consider lipid peroxidation, which can lead to the degradation of food flavor and potential damage to biological systems.

### 4.1 Analysis of Food Lipids

Analysis of lipids in food is crucial for determining nutritional value and constituting components, so evaluating the quality of food, promoting and explaining the effects of fats and oils on the functionality of food, and developing tailor‐made foods designed for specific applications. Proper sampling and storage of samples are crucial in food lipid analysis, as well as in all other forms of chemical analysis, to ensure accurate and reliable results (Shahidi et al. 2017). It is necessary to perform an extraction process before analyzing any lipid in food to separate the lipid components from proteins, amino acids, carbohydrates, and water components quantitatively (Nichols et al. 2011a). Extraction is a key step in most of the methods employed for testing lipids. Therefore, it is essential to take care during the lipid extraction process to avoid any conversion of lipids into other forms. Extraction should be performed as quickly as possible while minimizing oxidation and any subsequent changes. On the other hand, before the extraction itself, food samples need to be prepared for efficient extraction. For this purpose, it is possible to carry out pretreatment such as reducing the size of the samples, drying them, and subjecting them to hydrolysis (Shahidi et al. 2017; Nichols et al. 2011a; Zamuz et al. 2022). Several official procedures for solvent extraction can be used on a variety of food samples: Association of Official Analytical Collaboration (AOAC) International, American Oil Chemistry Society (AOCS), American Association for Clinical Chemistry (AACC), International Union of Pure and Applied Chemistry (IUPAC), International Organization for Standardization (ISO). The choice of suitable methods depends on the specific characteristics of the food and the investigated lipids. Nevertheless, each available method has its own set of advantages and disadvantages. All these official methods depend on the interaction between the solvent and lipids; hence their effectiveness is determined by the polarity of the lipids and solvents. Polar lipids, such as glycolipids, sphingolipids, and phospholipids, have a higher solubility in polar organic solvents like alcohols. On the contrary, nonpolar lipids, such as triacylglycerols and cholesterol esters, have a higher solubility in nonpolar organic solvents such as hexane, chloroform, ethyl ether, and petroleum ether (Shahidi et al. 2017; Zamuz et al. 2022). The methods recommended by the official procedures and other conventional methods are also used to obtain lipids from foods by solvent extraction. One approach involves employing a single solvent, such as the Goldfish, Foss‐Let, Soxhlet, or Rose‐Gottlieb procedures. Another approach involves utilizing a combination of two or more solvents, as shown in methods like Folch, Bligh, and Dyer (Shahidi et al. 2017; Zamuz et al. 2022). Pressurized fluid extraction (PFE), pressurized liquid extraction (PLE), or accelerated solvent extraction (ASE) have been developed to improve the efficiency of conventional solvent extraction by using classical solvent systems under varying extraction parameters like temperature, pressure, and volume (Shahidi et al. 2017). The major disadvantages of conventional solvent extraction methods include the degradation of bioactive compounds at high temperatures, the toxicity of solvents, the need for various analytical steps, significant solvent consumption, and a long extraction time (Ozogul et al. 2018). In recent years, novel methods such as microwave‐assisted, ultrasound‐assisted extraction, and supercritical fluid extraction, which are relatively new, innovative, and environmentally friendly methods, have begun to be used in oil extraction to overcome traditional solvent extraction methods' drawbacks.

The properties of extracted lipids are important to define the quality. There are several methods to analyze the lipid properties, like iodine value (IV), acid value, oxidative stability, refractive index, saponification value, and solid fat index. IV gives information about the degree of unsaturation of lipids. The acid value or neutralization value of lipids defines the unbounded free fatty acid (FFA) content as mg KOH required to neutralize 1 g of lipid (Nichols et al. 2011b; Shahidi et al. 2017). While refined oils mostly contain very low FFA, crude oils contain a significant amount. So, this parameter could serve as a measure of the oil's quality (Nichols et al. 2011b). Oxidative stability provides information about the autoxidation of unsaturated lipids. As a result of autoxidation reactions, primary products (hydroperoxides) and secondary products (aldehydes, ketones, alcohols, and hydrocarbons) are formed, which affect the quality of the lipid (Li et al. 2023; Shahidi et al. 2017). The refractive index gives information about the purity of oils and is measured with a refractometer. Since the refractive index varies with density, it depends on the unsaturation rate. As the unsaturation ratio increases, the refractive index increases (Nichols et al. 2011b). The saponification value is related to the average molecular weight of lipids. It is the amount of KOH (mg) required to saponify 1 g of lipid sample. The solid fat index is an empirical representation of the ratio of liquids in fat at a specific temperature. Dilatometry is used to determine how much solid fat melts and the volume of the sample increases. Also, low‐resolution pulse NMR and FTIR determine the solid fat content (Shahidi et al. 2017). In addition, some physical properties of oils, such as density, surface tension, viscosity, polarity, and melting point, are used to obtain information about their chemical structures and functional groups (Nichols et al. 2011b).

### 4.2 Novel Lipid Characterization Techniques

#### 4.2.1 Chromatographic Methods

Lipids contain different chemical compounds such as FAs, trans FAs, tocopherols, sterols, and pigments. Preparative or analytical separation techniques are used in the characterization analysis of lipids. Column chromatography, TLC, GC, HPLC, and supercritical fluid chromatography (SFC) are chromatographic techniques used in the characterization of lipids (Shahidi et al. 2017).

Column chromatography is frequently used to separate extracted lipids into fractions. This method is grouped as liquid–liquid (partition), solid–liquid (adsorption), and ion exchange chromatography. Solid–liquid chromatography is based on adsorption between the stationary phase (solid) and the mobile phase (liquid), depending on the polarity of the lipid components. Elution of the column with solvents of increasing polarity allows the separation of lipid fractions. When compared to classical column chromatography, commercial columns prepacked with stationary phases are used for lipid class separation, requiring less solvent, time, and packing material (Shahidi et al. 2017). Separation of complex lipids with prepacked inverted columns has been practiced for a long time (Figlewicz et al. 1985). For example, Mu et al. (2016) used argentated silica gel column chromatography to purify DHA and EPA.

TLC is a basic method used to determine lipid class profiles. TLC is not sufficient to classify compounds individually and is used together with spectroscopic methods. Tietel (2024) used two‐dimensional TLC to separate algal lipid extracts. The lipid class identification was validated using liquid chromatography‐tandem mass spectroscopy. As a result, it was found that the proposed two‐dimensional TLC method was successful in the classification of extracted algal lipids. The advantage of the TLC method is that it is simple and fast. Recently, high‐performance TLC (HPTLC), which has higher repeatability and resolution, has been used. In HPTLC applications, device use is important in the processing, drying, and staining of the plate. Separation of complex samples is visible in this technique and has an accuracy comparable to HPLC. Additionally, less solvent is used compared to HPLC, and it is more environmentally friendly (Engel and Schiller 2021). Kapoor et al. (2023) identified and quantified the major neutral and polar lipids of the milk fat globules using HPTLC. In the study, the advantages of the TLC method, such as speed, low need for solvent, and low cost, were emphasized.

GC is frequently used in the quantitative analysis of FAs. Before analysis, FAs must be derivatized into fatty acid methyl esters (FAME), which are more volatile nonpolar derivatives of polar carbonyl groups. The conversion to FAME is carried out in conjunction with flame ionization detection (FID). FID lacks information on molecular mass or structural characteristics for differentiating FAs. Full chromatographic resolution is needed for reliable quantification of all analytes of interest. In recent years, the use of MS together with GC enables the quantitative evaluation of FAMEs (Quehenberger et al. 2011). GC separation in conjunction with MS identification of lipid oxidation products is also used (Luo et al. 1995). Several derivatization methods are applied to FAs, such as acidic derivatization (hydrochloric acid, acetyl chloride, sulfuric acid) and basic derivatization (sodium methoxide). The column is another important point in GC analysis. FAs with varying chain lengths, saturation levels, double bond positions, and cis or trans isomers can be effectively separated with different columns. For FA analysis in biological samples, high‐polarity columns like the HP‐88 column (88% cyanopropyl aryl‐polysiloxane), DB‐FFAP column (nitroterephthalic acid‐modified polyethylene glycol), and SLB‐IL series columns (ionic liquids) are frequently employed (Chiu and Kuo 2020).

HPLC methods are used in the quantitative analyses. HPLC analyses are not limited to only volatile or thermally stable samples. The mobile phase used in the analysis is in liquid form, flowing through a column containing the stationary phase. Organic solvents (methanol and/or acetonitrile) are generally used as the mobile phase and pass through the column at high pressure (Zamuz et al. 2022). Column elution can be done in an isocratic, gradient, or stepwise manner and can be continuously monitored via a flow detector that is insensitive to flow rate, temperature, and composition of solvent (Shahidi et al. 2017). Various detectors are used in HPLC analyses. UV–visible detectors are the most commonly used and cost‐effective type. However, evaporative light scattering detectors (ELSD) and flame ionization detectors (FID) are used to determine saturated and unsaturated lipid mixtures (Moreau 2009). Recently, Yu et al. (2022) successfully determined the phospholipids of krill oil by a normal‐phase HPLC system with ELSD. Moreover, the use of mass spectrometric (MS) detectors has been widely used due to the high accuracy of mass fragmentation spectra (Nichols et al. 2011a).

The SFC technique has many advantages, such as analyzing FFAs and other lipids without a derivatization step and being studied at low temperatures. Since using nonpolar CO_2_ as the eluent is not suitable for polar lipid analysis, the addition of small amounts of organic solvent increases the solvent effect of the mobile phase (Donato et al. 2017). Packed and capillary columns and ELSD, FID, UV, MS, and FTIR detectors can be used for SFC (Shahidi et al. 2017). Lee et al. (2013) analyzed a polar lipid mixture containing phospholipids, lysophospholipids, and sphingolipids with supercritical fluid chromatography/tandem mass spectrometry (SFC/MS/MS) in 6 min. In another attempt, Tang et al. (2021) characterized 250 lipid species of milk samples by ultra‐HP SFC coupled to electrospray ionization (ESI) quadrupole time‐of‐flight MS.

#### 4.2.2 Spectroscopic Methods

Spectroscopic techniques like ultraviolet‐visible (UV–Vis), IR, NMR, and mass spectroscopy (MS) are widely used for the characterization of food lipids, with NMR and MS being the most commonly used methods. UV–Vis spectroscopy is the technique for identifying and quantifying food lipids that are less commonly used but have specific applications. In UV–Vis spectroscopy, light waves in the 100–750 nm range are used. Functional groups such as carbonyl and nitro groups with double, triple, or conjugated double bonds have considerable absorption in the UV or visible spectrum at specific wavelengths and molar extinction coefficients (Shahidi et al. 2017; Mishra 2022). UV–Vis spectroscopy is commonly used for the qualitative and quantitative analysis of carotenoids and related substances, as well as the analysis of oxidation products such as conjugated diene, triene, peroxide value, *p*‐anisidine value, and thiobarbituric acid value (Cirillo et al. 2017; Mishra 2022; Pike 2024). Furthermore, non‐destructive UV–Vis detectors used in HPLC measure the absorption of UV or Vis light by compounds eluted from the column (Zamuz et al. 2022).

IR spectroscopy uses the vibrations of atoms and molecules to analyze IR spectra, so providing evidence of molecular structure by examining the frequency of the normal mode of vibration (Mishra 2022). The IR spectrum of oil yields important information on the composition and functional groups of the lipid, as well as any impurities present (Shahidi et al. 2017). The mid‐region spectrum of oils and extracted lipids from foods provides information on oxidation levels and composition of oxidation products, enabling qualitative and quantitative analysis. Most information on oxidation products, including carbonyl compounds and trans isomers, is obtained from spectrum wavelengths ranging between 900 and 1745 cm^−1^ (Bartosz and Kołakowska 2011). FTIR spectroscopy is commonly used as a valuable analytical tool for quantifying the amount of total trans FAs (trans fat) in edible fats, oils, and extracted fats. The quantification of total trans fat using FTIR spectroscopy relies on the measurement of the C〓H out‐of‐plane deformation band at 966 cm^−1^, which is specifically associated with the absorbance of FAs individual trans double bonds (Srigley and Mossoba 2017). Additionally, the FTIR spectrometer measures parameters like IV, saponification value, and FFAs (Shahidi et al. 2017). Near‐infrared reflectance spectroscopy (NIRS) uses the overtone bands of hydrogen, oxygen, and carbon as a source of information in the wavelength range of 780–2500 nm. NIRS has been used frequently in oil analysis for purposes such as adulteration, saponification number, acid value, IV, peroxide value, sterols, and FA quantification in extra virgin olive oils and geographical authentication (classification and origin) (Rao et al. 2009; Haruna et al. 2022). The acid value and peroxide value of perilla seed oil were measured under various storage conditions and time intervals using NIRS (Hong et al. 2017). Raman spectroscopy is preferable to IR spectroscopy due to its greater sensitivity in detecting double‐bonded structures, specifically C〓C, which is weak in the IR spectrum (Kizil and Irudayaraj 2008). Raman spectroscopy is used by the oil industry, especially in the olive oil industry, to detect economically motivated adulteration, particularly extra virgin olive oil adulteration with refined or cheaper vegetable oils (Rodriguez‐Saona and Ayvaz 2024).

NMR spectroscopy is widely used in quantitative and qualitative analysis of the molecular structure of lipids (Figure 5D). Compared to chromatographic methods, it is stated that it has advantages such as being non‐destructive and not requiring standards for quantification. Its sensitivity is low compared to MS (Alexandri et al. 2017). High‐resolution ^1^H NMR is used in quantitative and structural analyses of lipids. While high‐resolution ^13^C NMR is used to obtain structural information, ^31^P NMR is used in the analysis of lipid compounds containing phosphorus (Shahidi et al. 2017). ^13^C and ^31^P NMR are used to analyze FAs in lipid mixtures. Nieva‐Echevarría et al. (2014) performed the characterization and quantitative analysis of the hydrolysis products of triglycerides during lipolysis using ^1^H NMR. Additionally, researchers have examined the lipid profile of different foods by ^1^H NMR. For example, Iberian dry‐cured hams (Pajuelo et al. 2022) and cheese (Haddad et al. 2022). In addition to the main components, minor components such as squalene and sterols in lipids and changes in lipid components after thermo‐oxidative degradation can also be determined by ^1^H NMR (Martínez‐Yusta et al. 2014, Table 2).

**TABLE 2:** Comparative analysis of selected characterization techniques for polysaccharides, proteins, and lipids.

Analytical method	Target	Resolution	Applications	Advantages	Limitations	References
1. Polysaccharides						
HPLC‐MS^n^	Oligosaccharide structures	High	Confirmation of sugar residues	High‐resolution identification of oligosaccharides	Complexity in data interpretation	Wefers et al. (2015)
High‐performance size exclusion chromatography (HPSEC)	Molecular weight distribution of pectin fractions	High	Determining molar mass and hydrodynamic properties	Helps understand the macromolecular structure of pectin	Limited ability to distinguish between polysaccharide types	Muhidinov et al. (2023)
High‐performance anion‐exchange chromatography with pulsed amperometric detection (HPAEC‐PAD)	Monosaccharide composition	High	Analysis of sugar components in pectin fractions	High sensitivity, specificity, and resolution	Requires specialized columns and reagents	Muhidinov et al. (2023), D'Agostino et al. (2024)
High‐performance gel‐permeation chromatography (HPGPC)	Molecular weight determination	High	Determines homogeneity and molecular weight	Effective for polysaccharides	Limited by standard calibration	Chen et al. (2015)
HPSEC‐RID	Molecular weight of extracted polysaccharides	High	Determining hydrodynamic volume	Reliable molecular weight estimation	Requires calibration standards	D'Agostino et al. (2024)
High‐performance liquid chromatography (HPLC)	Oligosaccharide composition	High	Structural characterization	Accurate quantification of components	Requires specialized equipment	Zheng et al. (2023)
Gas chromatography (GC)	Monosaccharide analysis	High	Identification of sugar components	High sensitivity for volatile compounds	Derivatization needed for non‐volatile compounds, requires sample hydrolysis	Fauziee et al. (2021), Chen et al. (2015)
Gel‐permeation chromatography‐refractive index‐multiangle laser light scattering (GPC‐RI‐MALS)	Molecular weight determination	Moderate to high	Polysaccharide structural characterization	Provides insight into polysaccharide polydispersity and molecular distribution	Requires sophisticated instrumentation	Huang et al. (2021)
Ion chromatography (IC)	Monosaccharide composition	High	Characterization of polysaccharides	Helps determine acidic polysaccharides with predominant glucuronic acid	Limited to detecting specific sugars	Huang et al. (2021)
Fourier‐transform infrared spectroscopy (FTIR)	Functional group analysis, degree of esterification	Moderate to high	Identifies structural features, identifying molecular bonds and starch modifications	Simple and non‐destructive, identifies esterified and carboxylated groups in pectin, confirms presence of acidic polysaccharose, pyranose rings, provides insights into molecular interactions and chemical modifications	Lower specificity compared to other methods, overlapping spectra can limit resolution of complex molecules, limited to surface functional groups	Chen et al. (2015), Muhidinov et al. (2023), Tang et al. (2021), Huang et al. (2021), Shrivastava et al. (2024)
Nuclear magnetic resonance (NMR) spectroscopy	Structural confirmation of isolated oligosaccharides, molecular structure determination	High	Identification of sugar arrangements, structural elucidation of complex polysaccharides	Provides detailed molecular characterization, identifies acetylation and branching, provides detailed atomic‐level insights	Requires pure sample and precise conditions, weak signals for high‐molecular weight samples, expensive and time‐consuming	Tang et al. (2021), Muhidinov et al. (2023)
Solid‐state NMR spectroscopy	Polysaccharide dynamics and interactions	High	Structural analysis of plant cell walls	Provides molecular insights into carbohydrate behavior	Requires specialized equipment and expertise	Kirui et al. (2021)
^1^H‐NMR	Chemical structure, degrees of acetylation and methylation	High	Structural characterization	Quick and efficient evaluation of polysaccharides	Potential interference from complex matrices	D'Agostino et al. (2024)
Two‐dimensional magic‐angle‐spinning solid‐state nuclear magnetic resonance (MAS SSNMR)	Polysaccharides (cellulose, hemicellulose, pectin)	High	Investigation of intermolecular interactions between cell wall components; structural characterization of cell wall proteins	Provides molecular‐level insights into cell wall architecture; enables direct spatial proximity measurements; preserves native molecular interactions	Requires isotopic labelling for sensitivity, limited in detecting highly mobile or disordered components; signal overlap may complicate analysis	Wang et al. (2012)
Scanning electron microscopy (SEM)	Micromorphology of conjugates, surface morphology, granule shape	High	Studying starch granule size and morphology, observes size and shape variations of glycosylation products	Offers detailed visualization of structural changes, shows surface morphology transformations due to Maillard reaction	Requires sample drying, which may alternative structures, limited field of view, requires coating	Tao et al. (2024), Shrivastava et al. (2024)
X‐ray diffraction (XRD)	Crystallinity, phase composition	High	Determining starch granule structure	Useful for analyzing crystalline regions and polymorph types	Requires high‐quality sample preparation	Shrivastava et al. (2024)
Circular dichroism (CD) spectroscopy	Secondary structural changes in SPI/SP‐GP	High	Monitors changes in α‐helix and β‐sheet structures	Provides insights into molecular stability and protein unfolding	Limited in detecting tertiary structural alterations	Tao et al. (2024)
Confocal laser microscopy	Emulsion droplet characteristics	High	Evaluates emulsion stability and uniformity	Distinguishes protein‐polysaccharide interactions affecting emulsification	Requires fluorescent dyes that may interact with sample	Tao et al. (2024)
Laser particle size analyzer	Emulsion particle size and span	Micrometer‐scale resolution	Analyses particle size distribution	Confirms the effectiveness of glycosylation in creating stable emulsions	Limited in detecting structural changes in individual molecules	Tao et al. (2024)
Differential scanning calorimetry (DSC)	Thermal properties, gelatinization	High	Assessing gelatinization and thermal behavior	Helps determine thermal stability and processing conditions	Requires precise calibration, sensitivity to impurities	Shrivastava et al. (2024)
Zeta potential measurement	Surface charge of emulsions	High precision charge analysis	Determines electrostatic stability of emulsions	Higher Zeta potential indicates improved emulsion stability	Does not assess molecular interactions beyond charge measurements	Tao et al. (2024)
2. Proteins						
Nuclear magnetic resonance (NMR)	Small to medium‐sized proteins, biomolecular dynamics	Variable (~1–5 Å) [Lower resolution compared to X‐ray]	Structural determination of flexible molecules, studying protein dynamics	Can study molecules in native states, captures dynamic information	Limited for large macromolecules, requires high sample concentration (mM level), limited structural resolution	Chari and Stark (2023)
Cryo‐electron microscopy (cryo‐EM)	Biological macromolecules, protein complexes	High [3–5 Å (average), potential for atomic resolution (< 1 Å)]	Structural determination of biomolecules, visualization of protein aggregates	Enables visualization of proteins, complements X‐ray crystallography and NMR; suitable for large and flexible molecules, captures multiple conformational states, works on large molecules, avoids crystallization challenges, near‐atomic resolution	Optical aberrations, atomic resolution constraints, biochemical sample preparation challenges, requires large proteins (> 150 kDa), high sample homogeneity, resolution gap for pharmaceutical research (~2 Å needed), expensive and requires expertise	Chari and Stark (2023), Nogales and Scheres (2015), Housmans et al. (2023)
X‐ray crystallography	Proteins (~10–150 kDa), crystallized proteins	High [Near‐atomic resolution (~2 Å)]	Structural biology research, atomic‐level structure determination	High‐resolution, dominant technique for protein structure analysis, well‐established methodology	Requires crystallization, which may be difficult for some proteins	Chari and Stark (2023)
Differential scanning calorimetry (DSC)	Protein (stability)	High	Thermal stability analysis for drug formulations	Precise thermodynamic measurements	Requires large sample amounts	Housmans et al. (2023)
Dynamic light scattering (DLS)	Protein (aggregation state)	Low‐medium	Size distribution of protein aggregates	Rapid and easy measurement	Cannot distinguish between different aggregates	Housmans et al. (2023)
Size exclusion chromatography‐MALS	Protein (molecular weight and oligomerization)	Moderate	Determining aggregation state and molecular mass	High sensitivity	Limited by column resolution	Housmans et al. (2023)
Circular dichroism (CD)	Secondary structure	Moderate	Structural characterization of proteins	Useful for secondary structure estimation	Low resolution for tertiary structure analysis	Housmans et al. (2023)
Fourier‐transform infrared spectroscopy (FTIR)	Protein secondary structure	Moderate	Secondary structural analysis	Can be applied to complex samples	Interference from water absorption	Housmans et al. (2023)
Atomic force microscopy (AFM)	Surface topography of protein aggregates	High	Structural analysis of aggregates	Can resolve single molecules	Limited to surface structures	Housmans et al. (2023)
Gel electrophoresis	Protein separation	Moderate	Identification and purity analysis	Standard technique in many labs	Limited resolution for large protein complexes	Housmans et al. (2023)
Radial immunodiffusion (RID)	IgG (quantification)	Moderate	Detection in complex matrices like milk	Accurate and repeatable results	High investment and long incubation time	Gao et al. (2022)
Enzyme‐linked immunosorbent assay (ELISA)	IgG (detection)	Moderate	High‐throughput IgG quantification	Low cost, widely used	End‐point measurement prevents real‐time monitoring	Karl and Staufenbiel (2017), Kessler et al. (2019)
Surface plasmon resonance (SPR)	IgG (binding interaction)	High	Label‐free biosensing	Real‐time analysis	Requires expensive materials and complex instruments	Crosson and Rossi (2013)
Liquid chromatography–tandem mass spectrometry (LC–MS/MS)	IgG, lactoferrin, α‐lactalbumin	High	Protein detection and quantification	High specificity, sensitivity, reliability	Requires advanced instrumentation	Li et al. (2019)
Deep learning‐based prediction (AlphaFold2)	Single protein chains	High	Protein structure modeling, drug discovery	High accuracy	Requires large datasets, limited for complexes	Wodak et al. (2023)
Ab initio docking methods	Protein–protein interactions	Moderate	Predicting native protein complexes	Fast Fourier‐Transform (FFT) methods enable rapid docking	Struggles with flexible proteins, requires refinement	Wodak et al. (2023)
Template‐based docking	Homologous protein complexes	High	Modeling complexes from known structures	High accuracy if homologous templates exist	Limited to proteins with known homologous complexes	Wodak et al. (2023)
Molecular mechanics & force fields (CHARMM, Amber, ECEPP)	Protein (stability, folding)	Moderate	Computational modeling of protein structures	Accounts for covalent, noncovalent, and electrostatic interactions	Struggles with solvation effects, convergence issues	Wodak et al. (2023)
3. Lipids						
Thin‐layer chromatography (TLC)	Lipid classes	Low	Separation of lipid components, traditional lipid separation	Simple and cost‐effective	Low sensitivity, limited identification capability, Limited resolution and quantification	Della Corte et al. (2015), Carrasco‐Pancorbo et al. (2009), Navas‐Iglesias et al. (2009)
Raman spectroscopy	Lipid molecular structure, unsaturation levels	Moderate	Lipid oxidation studies, food quality assessment	Non‐invasive, label‐free analysis	Lower sensitivity, interference from biological matrices	Li, Han, et al. (2014)
Near‐infrared (NIR) spectroscopy	Organic versus non‐organic egg classification	Moderate	Authentication of egg production systems	Non‐destructive, rapid analysis	Limited specificity	Hoffman et al. (2022)
Fluorescence excitation spectroscopy	Carotenoid signatures in eggs	Moderate	Differentiation of organic and non‐organic eggs	Can detect luminescence properties of egg yolks	Requires specialized excitation sources	Wohlers et al. (2022)
Nuclear magnetic resonance (NMR)	Structural characterization of lipids	Moderate	Lipid metabolism studies, authentication of conventional vs. organic eggs	Provides structural insights, non‐destructive, comprehensive structural insights Simple sample preparation	Lower sensitivity compared to MS, requires large sample amounts	Zhang et al. (2022), Ackermann et al. (2019), Harrieder et al. (2022)
Gas chromatography–mass spectrometry (GC–MS)	Fatty acid composition	High	Lipid profiling in food matrices, determination of saturated and unsaturated fatty acids in milk samples, identification of lipid profiles in grape seed oil	High separation efficiency, sensitive quantification, effective for volatile lipid analysis, suitable for identifying individual fatty acids	Requires hydrolysis and derivatization, limiting structural analysis, limited to volatile lipids	Zhang et al. (2022), Della Corte et al. (2015), Yang et al. (2021)
High‐performance liquid chromatography (HPLC)	Phospholipids, sphingolipids, glycerolipids	High	Comprehensive lipid profiling, metabolic studies	High resolving power, reproducibility	Time‐consuming, requires complex sample preparation	Li, Han, et al. (2014)
Ultra‐high performance liquid chromatography (UHPLC)	Lipid profiling	High	Separation of lipid species	Enhanced sensitivity and selectivity	Requires specialized columns and solvents	Della Corte et al. (2015)
UHPLC–MS/MS (Ultrahigh‐performance liquid chromatography tandem mass spectrometry)	Lipid profiling, including glycerophospholipids, ceramides, and galactolipids	High	Comprehensive lipid analysis in grapes and maturation studies	Enables extensive lipid profiling with semi‐quantification of 412 compounds; robust method suitable for plant lipid research	Requires specialized equipment and expertise; complex sample preparation	Masuero et al. (2021)
UHPLC‐QE Orbitrap/MS/MS	High‐resolution lipid identification	Ultrahigh	Mushroom species differentiation	High precision and sensitivity for lipid classification	Expensive instrumentation, data complexity	Yang et al. (2021)
Ultrahigh‐performance liquid chromatography‐Quadrupole time‐of‐flight mass spectrometry (UHPLC‐Q‐TOF‐MS)	Lipid profiling, molecular species identification	High	Comparative lipidomics analysis of human milk and infant formulas	High sensitivity, rapid analysis, minimal sample preparation	Complexity in lipid structure may require extensive optimization	Zhang et al. (2022)
Liquid chromatography–Mass spectrometry (LC–MS)	Lipid composition of pecan	High	Lipid profiling, food authentication, Identification of TG profiles in plant‐based and bovine milk	Provides detailed lipid profiles, identification of key fatty acids, provides full molecular structure of lipids, high sensitivity and quantitative analysis	Requires specialized equipment, time‐intensive sample preparation, weak UV absorbance for lipids	Zhao et al. (2023), Lísa and Holčapek (2015), Wei et al. (2013), Adnani et al. (2012), Salghi et al. (2014), Russo et al. (2013), Yang et al. (2021)
LC–MS/MS	Free fatty acids, glycerolipids, glycerophospholipids, triterpenoids	High	Differentiation of lipid composition in various grape tissues & cultivars	Provides detailed lipidome characterization; identifies lipid markers; enhances understanding of lipid impact on fermentation & wine sensory profile	Limited to lipidomic analysis; does not integrate genetic or environmental influences	Pérez‐Navarro et al. (2019)
LC‐ESI‐MS/MS (Liquid chromatography electrospray ionization tandem mass spectrometry)	Fatty acids, sterols, glycerolipids, glycerophospholipids, sphingolipids	High	Quantitative profiling of grape lipids	Rapid and sensitive analysis, minimal sample preparation, broad lipid coverage	Complexity of lipid structure may require extensive optimization, potential matrix effects	Della Corte et al. (2015)
UPLC‐TIMS‐Q‐TOF‐MS	Lipid profiling	High	Comprehensive characterization of lipids in meat	Enhanced lipid identification via ion mobility separation	Requires specialized equipment and expertise	Martakos et al. (2024)
UPLC‐Q‐exactive orbitrap MS	Diverse lipid species in food products	Ultrahigh	Milk authentication, lipid biomarker discovery	Identifies lipid biomarkers for fraud detection	Requires high‐end instrumentation	Li et al. (2017)
Ultra‐performance liquid chromatography–mass spectrometry (UPLCMS/MS)	Lipidomic profiling	High	Identification of lipid composition in eggs	Sensitive, detects a wide range of lipid species	Requires sophisticated instrumentation	Chin et al. (2023)
Supercritical fluid chromatography (SFC) coupled to Q‐TOF‐MS	Triacylglycerols (TAGs)	High	Identification of TAGs in human milk and infant formulas	Comprehensive lipid profiling, metabolic studies	Requires specialized equipment, complex sample preparation	Tu et al. (2018)
Ion mobility‐mass spectrometry (IM‐MS)	Isobaric lipid compounds	Very high	Complex biological lipid samples	Resolves mobility differences based on lipid structures	Requires specialized equipment	Milic et al. (2015), Leng et al. (2015), Lintonen et al. (2014)
Electrospray ionization (ESI) MS	Lipid profiling, molecular species identification	High	Shotgun lipidomics, biomarker discovery	High sensitivity, rapid analysis, minimal sample preparation	Difficulty in distinguishing isomers, matrix effects	Li, Butka et al. (2014)
Matrix‐assisted laser desorption Ionization (MALDI) MS	Lipidomics imaging, lipid–protein interactions	High	Disease biomarker discovery, tissue imaging	High spatial resolution, simultaneous detection of lipids and proteins	Limited quantification capability, requires optimized matrix selection	Li, Han, et al. (2014)

MS and MS/MS identify compounds based on their mass‐to‐charge ratios. To draw the mass spectrum, the samples are ionized and positively charged fragments are created. Different techniques can be used in ionization: atmospheric pressure chemical ionization (APCI), fast atom bombardment (FAB), matrix‐assisted laser desorption/ionization linked to time‐of‐flight (MALDI‐TOF), and ESI. The ions are accelerated and subjected to magnetic or electrical fields and detected by the electron multiplier (Zamuz et al. 2022). MS is used together with GC and HPLC in the structural analysis of lipid molecules. In a study, the lipid content of coconut, andiroba, and castor seed oils was determined using GC–MS, ultra‐efficiency LC–MS, and easy ambient sonic‐spray ionization‐MS methods (Bataglion et al. 2014). In another attempt made in recent years, *Bischofia polycarpa* seed oil was extracted with several solvents and its phytosterol and squalene contents were determined by GC–MS (Wang, Su, et al. 2023). In another study, avocado oil was extracted by squeezing, aqueous, and supercritical carbon dioxide extraction techniques, and its lipids were analyzed. For this purpose, UPLC‐TOF tandem MS/MS was used. Also, the differential components of lipids were analyzed by orthogonal partial least squares‐discriminant analysis (OPLS‐DA), coupled S‐plot with variable significance in projection (VIP) (Liu et al. 2023).

### 4.3 Emerging Techniques

Lipidomics, driven by HRMS, has significantly advanced the understanding of lipidomes in biological systems and food science. Lipids influence nutrition, metabolism, food quality, and safety, with lipidomic profiling enabling comprehensive analysis across various bio‐resources (Chen et al. 2017). Masuero et al. (2021) developed an ultrahigh‐performance liquid chromatography tandem mass spectrometry (UHPLC–MS/MS) method for comprehensive lipid profiling in grapes. Key characterization techniques included mass spectrometry‐based lipidomics, leveraging ESI for high specificity and sensitivity (Masuero et al. 2021). Similarly, Della Corte et al. (2015) introduced a rapid LC–MS/MS method for lipid characterization in grapes, focusing on FAs, sterols, glycerolipids, glycerophospholipids, and sphingolipids. It highlights how liquid chromatography coupled with ESI tandem mass spectrometry (LC‐ESI‐MS/MS) enables efficient extraction, identification, and quantification of lipids with minimal sample preparation. The method was validated for 33 lipids, demonstrating high sensitivity, specificity, and reproducibility (Della Corte et al. 2015).

Martakos et al. (2024) developed an untargeted 4D lipidomics workflow for pork and chicken meat characterization, integrating microextraction with methyl tertiary‐butyl ether and methanol, followed by UPLC‐TIMS‐Q‐TOF‐MS analysis, which enhanced lipid identification through ion mobility separation. This method significantly improved resolution and selectivity, identifying 395 lipids across multiple classes, offering insights into phospholipids, glycerolipids, and n‐3 FAs in food composition. Yang et al. (2021) applied various lipid characterization techniques to differentiate wild edible mushrooms, using GC–MS for fatty acid analysis and LC–MS, particularly UHPLC‐QE Orbitrap/MS/MS, for high‐resolution lipid identification and quantification. Chemometric methods such as PCA and PLS‐DA facilitated species differentiation, while hierarchical clustering revealed lipid content similarities, advancing nutritional evaluation and food science applications. Chin et al. (2023) investigated chicken egg provenance through lipidomic profiling and machine learning, comparing isopropanol and MTBE extraction methods, with isopropanol proving more efficient. UPLC–MS/MS distinguished cage, barn, and free‐range eggs based on phosphocholine, phosphoethanolamine, and acylglyceride composition, while spectroscopic techniques like NIR, NMR, and fluorescence spectroscopy aided food authentication. Machine learning models, including OPLS‐DA and SVM, ensured accurate classification, supporting food fraud detection and provenance verification (Chin et al. 2023). The future of lipidomics in food science is promising, with high‐throughput platforms, enhanced MS accuracy, advanced imaging techniques, and stronger bioinformatics driving deeper lipid research and functional insights (Chen et al. 2017).

Foodomics leverages advanced omics technologies, including genomics, transcriptomics, proteomics, and metabolomics, to analyze food composition, authenticity, bioactivity, and safety, ultimately enhancing consumer health and confidence. For instance, Sidira et al. (2024) highlighted the role of proteomics, metabolomics, and lipidomics in meat characterization. Proteomics applies mass spectrometry to analyze proteins, supporting quality assessment and authentication. Metabolomics leverages LC–MS and NMR to examine small molecules, identifying biochemical variations and potential adulteration. Lipidomics, using HRMS, evaluates lipid composition to detect fraudulent modifications. These omic approaches enhanced insights into meat safety, processing, and quality, contributing to advancements in the food industry (Sidira et al. 2024).

Future applications of foodomics will expand into food safety, quality control, and nutritional research, with emerging trends such as transgenic food development, biomarker discovery, and omics‐based assessments of food bioactivity and health effects. Additionally, foodomics is expected to advance personalized nutrition, functional food innovation, and food traceability through sophisticated analytical techniques (Herrero et al. 2012). Advancements in artificial intelligence (AI) have significantly enhanced metabolomics analysis, leading to improved prediction accuracy. Metabolomics integrates high‐throughput analytical techniques with advanced bioinformatics, making it a vital tool in the post‐genomics era for functional genomics research. Its applications span multiple disciplines, including medicine, microbiology, and food science (Zhang et al. 2022), contributing to a deeper understanding of biological systems and metabolic processes.

## 5 Conclusions

Food is a rich source of complex biomacromolecules (i.e., polysaccharides, proteins, lipids), significantly influencing our eating experience due to their diverse functionalities. Prominent examples include starch, providing structure and browning in baked goods, and pectin in fruits, contributing to firmness. Beyond texture, food biomacromolecules serve as nature's energy stores (starch in grains) and play a crucial role in plant cell structure and human gut health. Scientists employ a sophisticated toolbox to unravel these intricate polymers. Spectroscopic methods like FTIR reveal the building blocks and linkages, while chromatographic techniques like HPSEC separate them by size, offering insights into branching patterns (amylose vs. amylopectin in starch). Thermal analysis techniques like DSC determine gelatinization temperature, which is crucial for food processing. By understanding these intricate structures and functionalities, food scientists can create innovative foods with specific textures and controlled nutrient release and even explore applications beyond the plate, like developing biodegradable food packaging materials using these remarkable polysaccharides. Nonetheless, challenges persist in bridging laboratory‐scale characterization with the complexity of real‐world food systems, where heterogeneity and multi‐component interactions complicate analysis. Integrative approaches that couple advanced techniques with computational modeling and machine learning are emerging as powerful strategies to address these challenges. Additionally, there is a growing need for high‐throughput, non‐destructive methods that can provide dynamic insights into biomacromolecules under industrially relevant conditions. Enhancing the characterization of proteins, lipids, and polysaccharides necessitates advancements in analytical methodologies, such as improved mass spectrometry and NMR spectroscopy, to achieve higher sensitivity and resolution in complex biomolecular studies. AI‐powered data analysis can streamline interpretation and support large‐scale screening efforts. For broader industry implementation, cost‐effective and scalable techniques must be developed to maintain precision while optimizing efficiency. Establishing standardized protocols across various platforms will improve reproducibility and foster interdisciplinary collaboration. Expanding multi‐omics strategies to concurrently examine these biomolecules will deepen understanding of their structural and functional roles in biological and industrial contexts.

## Author Contributions

**Seid Reza Falsafi:** project administration, supervision, writing – review and editing. **Julia Sebastian:** investigation (equal), validation (equal), visualization (equal), writing – review and editing (equal). **Rosana Colussi:** writing – review and editing (equal). **Lucas Ávila do Nascimento:** writing – original draft (equal). **Tansel Kemerli‐Kalbaran:** writing – original draft (equal). **Meral Yildirim‐Yalcin:** writing – review and editing (equal). **Ivan Adrian Garcia‐Galicia:** writing – original draft (equal). **Selin Sahin:** writing – original draft (equal). **Alma Delia Alarcon‐Rojo:** writing – review and editing (equal).

## Ethics Statement

This study does not involve any human or animal testing.

## Consent

Written informed consent was obtained from all study participants.

## Conflicts of Interest

The authors declare no conflicts of interest.

## Data Availability

Data will be made available on request.
